# *O*-Glycomic and Proteomic Signatures of Spontaneous and Butyrate-Stimulated Colorectal Cancer Cell Line Differentiation

**DOI:** 10.1016/j.mcpro.2023.100501

**Published:** 2023-01-18

**Authors:** K. Madunić, Y.M.C.A. Luijkx, O.A. Mayboroda, G.M.C. Janssen, P.A. van Veelen, K. Strijbis, T. Wennekes, G.S.M. Lageveen-Kammeijer, M. Wuhrer

**Affiliations:** 1 Center for Proteomics and Metabolomics, Leiden University, The Netherlands; 2 Department Chemical Biology and Drug Discovery, Utrecht Institute for Pharmaceutical Sciences and Bijvoet Center for Biomolecular Research, Utrecht University, Utrecht, The Netherlands; 3 Department Biomolecular Health Sciences, Utrecht University, Utrecht, The Netherlands

**Keywords:** CaCo-2, differentiation, omics integration, PGC-LC–MS/MS, glycomics, proteomics, ABC, ammonium bicarbonate, AP, alkaline phosphatase, B4GALNT2, β1–4-N-acetylgalactosaminyl transferase 2, BSM, bovine submaxillary mucin, CaCo-2, Cancer Coli-2, CRC, colorectal carcinoma, DPBS, Dulbecco’s PBS, ESI, electrospray ionization, FCS, fetal calf serum, GALNT, GalNAc transferase, GI, gastrointestinal, GMDS, GDP-mannose 4,6-dehydratase, GO, Gene Ontology, HCl, hydrochloric acid, HNF4A, hepatocyte nuclear factor 4-alpha, HNF1A, hepatocyte nuclear factor 1-alpha, ICC, ion charge control, MeCN, methyl cyanide, MS, mass spectrometry, PCA, principal component analysis, PGC, porous graphitized carbon, PNGase F, peptide N-glycosidase F, PVDF, polyvinylidene difluoride, TMT, tandem mass tag

## Abstract

Gut microbiota of the gastrointestinal tract provide health benefits to the human host *via* bacterial metabolites. Bacterial butyrate has beneficial effects on intestinal homeostasis and is the preferred energy source of intestinal epithelial cells, capable of inducing differentiation. It was previously observed that changes in the expression of specific proteins as well as protein glycosylation occur with differentiation. In this study, specific mucin *O*-glycans were identified that mark butyrate-induced epithelial differentiation of the intestinal cell line CaCo-2 (*Ca*ncer *Co*li-2), by applying porous graphitized carbon nano–liquid chromatography with electrospray ionization tandem mass spectrometry. Moreover, a quantitative proteomic approach was used to decipher changes in the cell proteome. It was found that the fully differentiated butyrate-stimulated cells are characterized by a higher expression of sialylated *O*-glycan structures, whereas fucosylation is downregulated with differentiation. By performing an integrative approach, we generated hypotheses about the origin of the observed *O*-glycome changes. These insights pave the way for future endeavors to study the dynamic *O*-glycosylation patterns in the gut, either produced *via* cellular biosynthesis or through the action of bacterial glycosidases as well as the functional role of these patterns in homeostasis and dysbiosis at the gut–microbiota interface.

## Highlights

• Butyrate induces changes in the CaCo-2 *O-*glycome and proteome upon differentiation.
• Differentiated cells show a higher expression of sialylated *O*-glycan structures.
• Terminal fucosylation shows lower expression in the differentiated cells.
• Butyrate induces changes in monosaccharide conversion and cell adhesion proteome.

## Graphical Abstract

**Figure:**
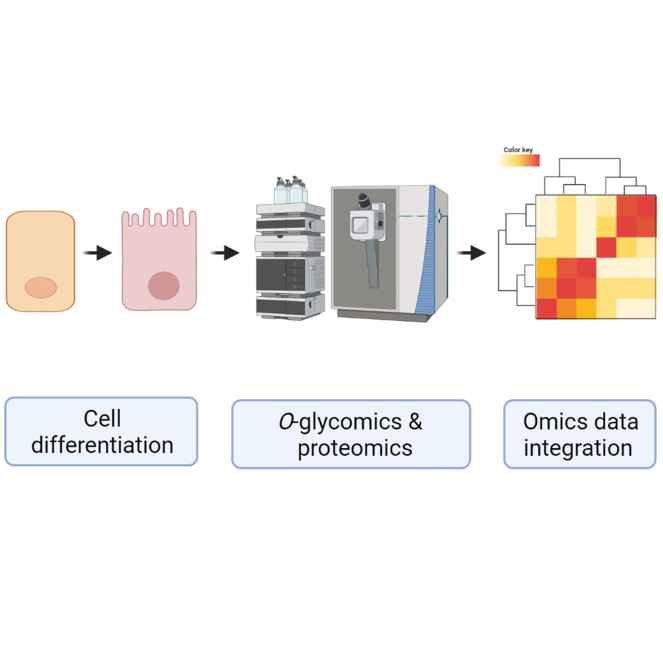

## In Brief

Bacterial butyrate is beneficial for intestinal homeostasis as the preferred energy source of intestinal epithelia, capable of inducing differentiation. Here, we describe glycomic and proteomic signatures of butyrate-induced epithelial differentiation of the intestinal cell line CaCo-2. We identified an upregulation of specific *O*-glycan signatures as well as specific proteins involved in cell metabolism, monosaccharide precursor biosynthesis, and cell adhesion. Using an integrative approach, we generated hypotheses about the origin of the glycosylation changes with differentiation.

The human gut microbiota is a complex ecology of a variety of different microorganisms. Among viruses, prokaryotes, and eukaryotes, bacteria are the most abundant inhabitants of the human gastrointestinal (GI) tract. By coevolution with the host, a symbiotic relationship has been formed between the GI tract and its bacteria (1, 2). The maintenance of homeostasis in the GI tract depends on the complex process of epithelial cell differentiation (3). Bacterial metabolites also play a functional role in the maintenance of this homeostasis (4). Butyrate, a short-chain fatty acid, is a bacterial metabolite produced by bacterial fermentation of dietary fibers and is known to have a beneficial effect on the intestinal homeostasis. Physiological concentrations of butyrate inhibit cell proliferation, induce differentiation, and increase the rate of apoptosis for a number of tumor cell types *in vivo* and *in vitro* (5, 6, 7, 8). Furthermore, butyrate is the main energy source of intestinal epithelial cells and capable of upregulating the gene expression of both secreted and membrane-linked glycoproteins including mucins (9, 10, 11, 12, 13). Notably, bacterial short-chain fatty acids, such as butyrate, are suspected to extend their effect outside the gut as they have been implicated as possible mediators of phenomena observed in the gut–brain axis (14).

The mucus layer covers the epithelial cells to prevent them from being in direct contact with the microbiota. The function of the mucosal layer as a barrier is mostly maintained by the gel-forming mucins, which are large and highly *O*-glycosylated proteins secreted by the epithelial cells (15). In addition, the mucus layer and especially its *O*-glycans represent an important nutrient source for the surrounding microbiota and thereby contribute to bacterial colonization in the human gut. Furthermore, epithelial cells express cell adhesion proteins such as cadherins, which mediate homophilic interactions between neighboring cells forming adherens junctions important for maintaining organized tissue architecture in the gut (16). A wide variety of oligosaccharide structures can be attached to these glycoproteins, and the composition of these structures can vary within cell types because of differential expression of glycosidases and glycosyltransferases (17).

Low differentiation and loss of cell–cell adhesion are a hallmark of cancer cells; therefore, further insights into the molecular signatures and key regulators of epithelial differentiation are key for understanding the pathophysiology of cancer. For example, E-cadherin glycosylated with β1–6-branched *N*-glycans changes its cell membrane expression (18) and stability of the adherens junctions (19) leading to impaired cell adhesion. Notably, the bacterial metabolite butyrate can induce a change in the expression of different genes through inhibition of histone deacetylation including specific glycogenes such as β-galactoside-α-2–6-sialyltransferase (ST6GAL1) (20, 21, 22, 23), which can influence the glycosylation of other cell adhesion glycoproteins and consequently their function.

In order to study cellular differentiation, we investigated the intestinal cell line, CaCo-2 (*Ca*ncer *Co*li-2), derived from a human colorectal carcinoma (CRC) in 1977. This well-established cell line can differentiate spontaneously or by exposure to butyrate into polarized cells with morphological and biochemical features of the mature colonic epithelium (24). Spontaneous differentiation of CaCo-2 cells has been studied previously, including transcriptomic, proteomic, and glycomic analysis (25, 26, 27, 28, 29, 30, 31, 32). The changes in *O*-glycosylation upon butyrate differentiation have yet to be characterized, and insights into these changes are crucial for a better understanding of the role of glycans in the gut homeostasis. The aim of the present study was to identify specific *O*-glycomic and proteomic signatures that define butyrate-induced epithelial differentiation in relation to spontaneous differentiation to gain insights into *O*-glycan signatures of colon cancer cell line differentiation derived from the gut epithelium.

## Experimental Procedures

### Chemicals and Reagents

Sodium borohydride, sodium chloride, Dowex cation-exchange resin (50W-X8), ammonium bicarbonate (ABC), TFA, Dulbecco’s PBS (DPBS), hydrochloric acid (HCl), and dl-DTT were purchased from Sigma–Aldrich. Ethanol (Reag. Ph. Eur) and bovine submaxillary mucin (BSM), type I-S, were purchased from Merck. Tandem mass tag (TMT)pro label reagents, 8 M guanidine hydrochloride, Dulbecco’s modified Eagle’s medium, 0.25% trypsin/EDTA, and fetal calf serum (FCS) were obtained from Thermo Fisher Scientific. Potassium hydroxide was obtained from Honeywell Fluka. Solid phase extraction bulk sorbent carbograph was obtained from Grace Discovery Sciences. HPLC SupraGradient acetonitrile (methyl cyanide [MeCN]) was obtained from Biosolve. Peptide *N*-glycosidase F (PNGase F) and complete EDTA-free protease inhibitor cocktail tablets were purchased from Roche Diagnostics. A 96-well PP filter plate was purchased from Orochem Technologies. MultiScreen HTS 96 multiwell plates (hydrophobic Immobilon-P polyvinylidene difluoride [PVDF] membrane) and 96-well PP microplate were obtained from Millipore.

### Cell Culture

Human colorectal adenocarcinoma CaCo-2 cells were grown in Dulbecco’s modified Eagle's medium (Gibco, Thermo Fisher Scientific) with 10% FCS. Cells were subcultured at 80% confluency and maintained at 37 °C in a humidified incubator with 5% CO_2_. At day 0, when cells reached full confluency, butyrate was added to the appropriate cells (at 2 mM final concentration in the cell culture flask). For the treated cells, butyrate was added to the medium at each medium exchange. Cells were grown in three separate culture flasks (three biological replicates) for both butyrate-treated and spontaneously differentiated group. On days 5, 7, 14, 21, and 24, cells were collected. Prior to harvesting the cells, medium was removed and adherent cells were washed twice with DPBS and trypsinized using 0.25% trypsin–1 mM EDTA. To stop the trypsin activity, medium (without FCS) in a ratio of 2:5 (trypsin:medium; v/v) was added, and cells were pelleted at 300*g* for 5 min. Cells were resuspended in DPBS and counted and aliquoted to ∼2.0 × 10^6^ cells per replicate and washed twice with 1 ml DPBS for 3 min at 100*g*. The supernatant was removed, and cell pellets were stored at −20 °C until further use.

### Phase-Contrast Microscopy

Phase images were acquired using phase contrast microscopy with 40× objective. Images were acquired through a CMEX 5000 Microscope camera.

### Alkaline Phosphatase Activity

Alkaline phosphatase (ALP) activity was measured in cell lysates using a colorimetric assay according to the manufacturer’s instructions (Abcam). The cells were harvested with 0.25% trypsin–1 mM EDTA, washed with PBS, and subjected to ultrasonication in the supplied assay buffer. The obtained cell homogenates were stored at −80 °C until assayed. Lysed cells at different growth days were incubated with 1.7 mM *p*-nitrophenylphosphate for 60 min at 25 °C. Absorbance readings were taken at 405 nm with a microplate reader (Fluorimeter FLUOstar Omega; BMG Labtech). Results are expressed as milliunits/milligram protein. One unit is defined as the activity that hydrolyzes 1 μmol of *p*-nitrophenylphosphate/min at 25 °C.

### Cell Lysis and *O*-Glycan Release

Three biological replicates from each time point were analyzed. Frozen cell pellets containing ∼2.0 × 10^6^ cells were resuspended in 100 μl of lysis buffer containing Tris–HCl, EDTA, sodium chloride, and protease inhibitor cocktail. The cells were lysed using a Branson sonicator rod at 1.5 output power, and 25 μl of the suspension (containing ∼5 × 10^5^ cells) were loaded onto the preconditioned PVDF membrane plate wells. BSM (10 μg) was blotted in three different wells of the same PVDF membrane plate. The denaturation as well as the *N*- and *O*-glycan release were performed as described previously (31, 33). Briefly, the proteins were denatured on membrane using guanidine hydrochloride and DTT at 60 °C. Upon removal of denaturing agents, the *N*-glycans were released by PNGase F digestion overnight at 37 °C and recovered in MQ water. A total of 2 units of PNGase F was added to each well of the PVDF membrane plate containing lysates from approximately 0.5 million cells. Upon recovery of the *N-*glycans, the *O*-glycans were released from the same wells by reductive β-elimination, using 50 μl of 0.5 M sodium borohydride in 50 mM potassium hydroxide incubating at 50 °C for 16 h. Samples were desalted by performing Dowex cation exchange resin (50W-X8) and graphitized carbon solid phase extraction in self-packed 96-well filter plates. The samples were dried after cleanup and stored at −20 °C until analysis.

### PGC-LC–MS/MS Analysis

The *O*-glycan samples were then reconstituted in 20 μl of MQ water, and 2 μl were injected for analysis. Analysis was performed using a PGC nano-LC Ultimate 3000 UHPLC system (Thermo Fisher Scientific) coupled to an amaZon ETD speed ion trap (Bruker Daltonics). The samples were loaded using 100% buffer A (10 mM ABC) at a loading flow of 6 μl/min on a custom-made trap column (size 30 × 0.32 mm) packed with 5 μm particle size PGC stationary phase from Hypercarb PGC analytical column (size 100 × 4.6 mm, 5 μm particle size; Thermo Fisher Scientific). Afterward, the *O*-glycans were separated at a 0.6 μl/min flow rate on a custom-made PGC column (100 × 0.1 mm, 3 μm particle size obtained from Thermo Fisher Scientific) by applying a linear gradient from 1% to 50% buffer B (MeCN, 10 mM ABC) over 73 min. During the procedures, a constant column temperature of 45 °C was maintained. To continue, the LC system was coupled to an amaZon ETD speed electrospray ionization (ESI) ion trap MS using the CaptiveSpray source (Bruker Daltonics) with an applied capillary voltage of 1000 V in negative-ionization mode. The drying gas (N_2_) flow rate was set to 3 l/min, and the temperature was set at 280 ˚C. The nebulizer gas pressure was kept at 3 psi. The nanoBooster bottle (Bruker Daltonics) was filled with methanol, as a dopant solvent (34). MS spectra were acquired in enhanced mode within a mass to charge ratio (*m/z*) range of 380 to 1850. The maximum acquisition time was set to 200 ms, the ion charge control (ICC) to 40,000, and the target mass of smart parameter setting was set to *m/z* 900. MS/MS spectra were generated by collision-induced dissociation of the three most abundant precursors, applying an isolation width of 3 Thomson. In addition, ICC was set to 150,000, and the fragmentation cutoff was set to 27% with a 100% fragmentation amplitude using the Enhanced SmartFrag option (30–120% in 32 ms). To integrate area under the curve for each individual glycan isomer, extracted ion chromatograms of the first three isotopes were used in Bruker Compass DataAnalysis software (version 5.0). Peaks were manually picked and integrated. Total area normalization was employed for relative quantification of *O*-glycan species. Identification of *O*-glycan species was performed by comparison with PGC retention time, MS/MS spectra, and the BSM standard.

### Quantitative Proteomics Using TMT Labeling

Cell lysis, digestion, and TMT labeling were performed as described previously (35). In short, ∼1.0 × 10^6^ CaCo-2 cells were lysed in SDS lysis buffer (SDS [5%], Tris–HCl [100 mM, pH 7.6]) at 95 °C for 4 min. Protein concentration was determined by Pierce bicinchoninic acid protein assay (Thermo Fisher Scientific). Around 100 μg of protein was used for subsequent reduction with 5 mM Tris(2-carboxyethyl)phosphine, alkylation with 15 mM iodoacetamide, and quenching with 10 mM DTT. Protein lysates were purified by methanol–chloroform precipitation. The resulting protein pellets were resuspended in 40 mM Hepes (pH 8.4) and digested with trypsin (10 μg) for 16 h at 37 °C. Peptide concentration was measured with Pierce bicinchoninic acid assay.

The 10 different conditions together with common reference samples were arranged in triplicate in three TMTpro 13-plex experiments. Of each peptide preparations, 10 μg was dissolved in 25 μl of Hepes buffer (40 mM, pH 8.4) and incubated with 40 μg of one of 13 amino-reactive TMTpro label for 1 h at ambient temperature. Excess TMT label was quenched by addition of 6 μl 5% hydroxylamine and incubation for 15 min at ambient temperature. Labeled peptide samples were then mixed and freeze-dried. TMT-labeled mixtures were dissolved in 1 ml 10 mM ABC and fractionated on 1 cc C18 SPE cartridges (Oasis HLB; Waters) using 5%, 10%, 15%, 20%, 25%, and 35% MeCN in 10 mM ABC.

TMT-labeled peptides were dissolved in water/formic acid (100/0.1 *v/v*) and subsequently analyzed twice by on-line C18 nanoHPLC MS/MS with a system consisting of an Ultimate3000nano gradient HPLC system (Thermo) and an Exploris480 mass spectrometer (Thermo). Fractions were injected onto a cartridge precolumn (300 μm × 5 mm, C18 PepMap, 5 μm, 100 A) and eluted *via* a homemade analytical nano-HPLC column (50 cm × 75 μm; Reprosil-Pur C18-AQ 1.9 μm, 120 A [Dr Maisch]). The gradient was run from 2% to 40% solvent B (20/80/0.1 water/acetonitrile/formic acid; *v/v/v*) in 120 min. The nano-HPLC column was drawn to a tip of ∼10 μm and acted as the electrospray needle of the MS source. The mass spectrometer was operated in data-dependent MS/MS mode for a cycle time of 3 s, with a higher energy collision dissociation at 30 V and recording of the MS/MS spectrum in the orbitrap, with a quadrupole isolation width of 1.2 Da. In the master scan (MS1), the resolution was 120,000, the scan range was 400 to 1500, at standard automatic gain control target at maximum fill time of 50 ms. A lock mass correction on the background ion *m/z* at 445.12 was used. Precursors were dynamically excluded after n = 1 with an exclusion duration of 45 s and with a precursor range of 20 ppm. Charge states 2 to 5 were included. For MS2, the first mass was set to 120 Da, and the MS/MS scan resolution was 45,000 at an automatic gain control target of 200% at maximum fill time of 60 ms.

In a postanalysis process, raw data were first converted to peak lists using Proteome Discoverer, version 2.2 (Thermo Electron) and submitted to the UniProt database (*Homo sapiens*, 20,596 entries), using Mascot, version 2.2.07 (www.matrixscience.com) for protein identification. Mascot searches were performed with 10 ppm and 0.02 Da deviation for precursor and fragment mass, respectively, and trypsin as enzyme. Up to two missed cleavages were allowed. Methionine oxidation and acetyl on protein N- terminus were set as a variable modification; carbamidomethyl on Cys, TMTpro on N terminus, and Lys were set as a fixed modification. Protein false discovery rate was set to 1%. Normalization was performed on total peptide amount.

### Experimental Design and Statistical Rationale

In this study, the *O*-glycome and proteome of CaCo-2 cell line were analyzed from three biological replicates. The samples were collected and analyzed from five different time points, starting from day 5, to days 7, 14, 21, and 24, postconfluence in two groups: spontaneous differentiation and butyrate-stimulated differentiation. Differences between groups and time points were tested using two-way ANOVA with significance level of α = 0.05 both for glycomics and proteomics datasets. Data analysis and visualization was performed using in-house developed “R’’ scripts. To enable use of principal component analysis, imputation of minimum positive number (0.0001) was performed.

## Results

### Cell Differentiation Assay

**Fig. 1:**
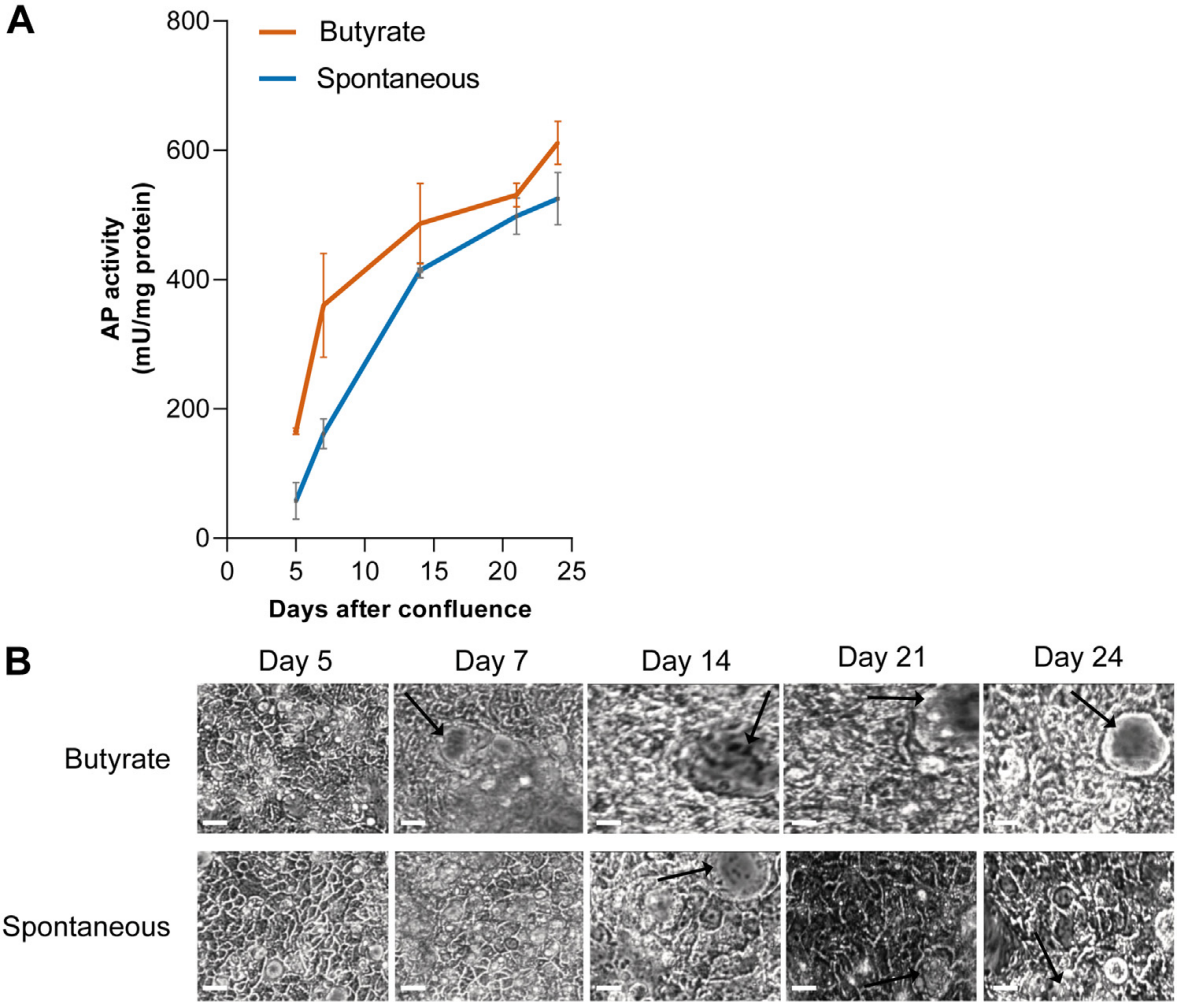
**Biochemical and morphological validation of CaCo-2 differentiation.***A*, alkaline phosphatase (AP) activity during CaCo-2 growth stimulated by 2 mM butyrate (*orange line*) or spontaneous differentiation (*blue line*). Standard deviation is indicated by error bars (n = 3). *B*, phase contrast images of CaCo-2 cells on culture dishes at different stages of growth. *Arrow*, dome-like structure. Original magnification, 40×. *White scale bar* represents 50 μm. CaCo-2, *Ca*ncer *Co*li-2.

CaCo-2 cells were analyzed at different time points during the process of spontaneous and butyrate-induced differentiation. Cells were proliferated to confluency, which is marked as day 0, after which cells start their differentiation. Differentiation of CaCo-2 cells was evaluated by a colorimetric assay measuring AP activity, which is a marker for late-stage differentiation, indicative for the presence of an established brush border (36). AP activity levels of both spontaneous differentiation and induced differentiation showed a continuing increase (Fig. 1*A*). A significantly higher level of AP activity for the induced differentiation was observed for days 5 and 7, compared with the spontaneous differentiation.

In addition to AP activity levels as a validation for differentiation, phase contrast images were taken of the apical surface (Fig. 1*B*) to observe differentiation-induced “dome” formation (37, 38). CaCo-2 cells imaged at different growth times started displaying dome formation at day 7 for the butyrate-treated cells, whereas the spontaneously differentiated cells showed dome formation later at day 14. After having established that CaCo-2 cells were differentiated, our next aim was to assess the effect of butyrate on the *O*-glycomic profile of these CaCo-2 cells.

### Glycomic Analysis

The analysis of *O*-glycans can be challenging in comparison to *N*-glycans, because of the lack of specific enzymes that can release the intact *O*-glycans from glycosylated proteins. In this study, we used a previously established protocol that allowed the chemical release of *O*-glycans from cell lysates *via* reductive β-elimination in 96-well plate format (31). *O*-glycans of the differentiating CaCo-2 cells were analyzed at days 5, 7, 14, 21, and 24 postconfluency. To further validate the robustness of this approach, full technical triplicates of the cell lysates containing 5 × 10^5^ cells from the same biological replicate (5 days, spontaneous differentiation) were processed and analyzed independently by porous graphitized carbon nano–liquid chromatography coupled to mass spectrometry (PGC-nanoLC–ESI–MS/MS). The mean relative area of the 11 most abundant *O*-glycan species and corresponding standard deviations are shown in Supplementary Information 1, supplemental Fig. S1*A*, demonstrating the low technical variability of the workflow. Parallel to this, the PGC-nanoLC–ESI–MS performance was assessed by releasing *O*-glycans from 10 μg of BSM standard and measuring them across 5 days, as illustrated in Supplementary Information 1, supplemental Fig. S1*B*. Overall, the method showed very good precision.

**Fig. 2:**
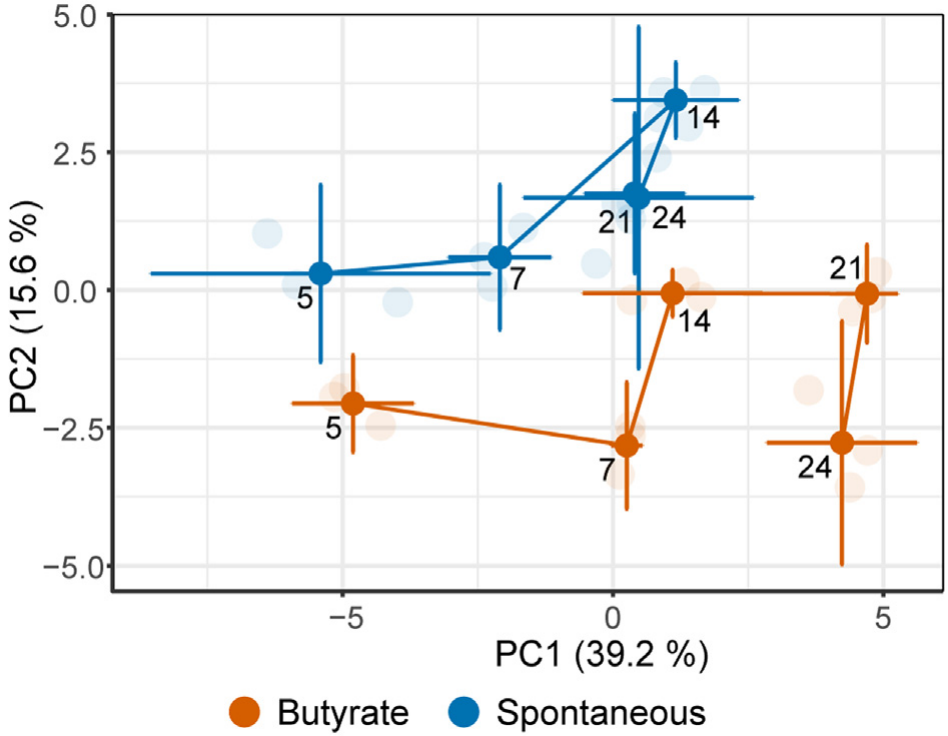
**The geometric trajectory visualization of glycomic changes with differentiation.** A distinction between the butyrate-stimulated group (*orange*) and spontaneous differentiation (*blue*) is observed in the PCA model based on relative abundance (%) of different *O*-glycans. The separation between different time points in the two groups is illustrated as a trajectory. The top two principal components (PCs) explain 54.8% of the variation within the data. The PCA scores from different biological replicates (*faded color*) were averaged to create the trajectory. PCA, principal component analysis.

To explore the *O*-glycomic profiles, a principal component analysis (PCA) was performed on relative abundances of individual glycans detected in the glycomic profiles (Supplementary Information 2, supplemental Tables S1 and S2). As illustrated in Supplementary Information 1, supplemental Fig. S2*A*, the model showed narrow clustering of most biological replicates, although substantial differences were observed within replicates for time points 24 days (butyrate) and 5 days (spontaneous). Different time points of differentiation were separated along the principal component 1 (39.2%). A clear separation was apparent between the butyrate-stimulated samples and spontaneously differentiated samples along principal component 2 (15.6%). A relative higher abundance of fucose containing *O*-glycans was found for the samples clustering in the upper left corner of the loadings plot, whereas sialylated *O*-glycans were enriched in the lower right part of the plot (Supplementary Information 1, supplemental Fig. S2*B*). Further analysis of the score plot (Fig. 2) showed that for days 5 and 7, both groups are different from one another as well as from days 14, 21, and 24, as no overlap is observed of the representations. In addition, when focused on the difference overtime within the butyrate and nonstimulated group (Fig. 2), the score plot indicates that the CaCo-2 *O*-glycome undergoes progressive changes when cells are grown from confluency to 7 days postconfluence, at which point these cells are not fully differentiated yet. Upon further differentiation, a distinct difference between the butyrate-stimulated group trajectory and the spontaneous differentiation trajectory can be observed. For the spontaneous differentiated CaCo-2 cells, the representations of days 14, 21, and 24 are close to one another. This indicates that the *O*-glycome appears to stabilize in the later differentiation phases. However, the butyrate-stimulated cells are different from each other between days 14, 21, and 24, which indicates that the butyrate-stimulated cells still undergo progressive changes in their *O*-glycome in the late differentiation phases. To support this visual interpretation and identify the glycosylation signatures changing with differentiation, two-way ANOVA was performed on relative abundances of individual *O*-glycans (Supplementary Information 2, supplemental Table S3). Specific *O*-glycans that show statistically significant changes with time (butyrate-stimulated *versus* spontaneous differentiation) are summarized in Supplementary Information 1, supplemental Fig. S3, Supplementary Information 2, and supplemental Table S4. The *O*-glycans that show a difference between the groups (butyrate-stimulated and spontaneous differentiation groups) are listed in Supplementary Information 2, supplemental Table S5. Moreover, the *O*-glycans that show a significant change with both butyrate stimulation and time are listed in Supplementary Information 2, supplemental Table S6.

**Fig. 3:**
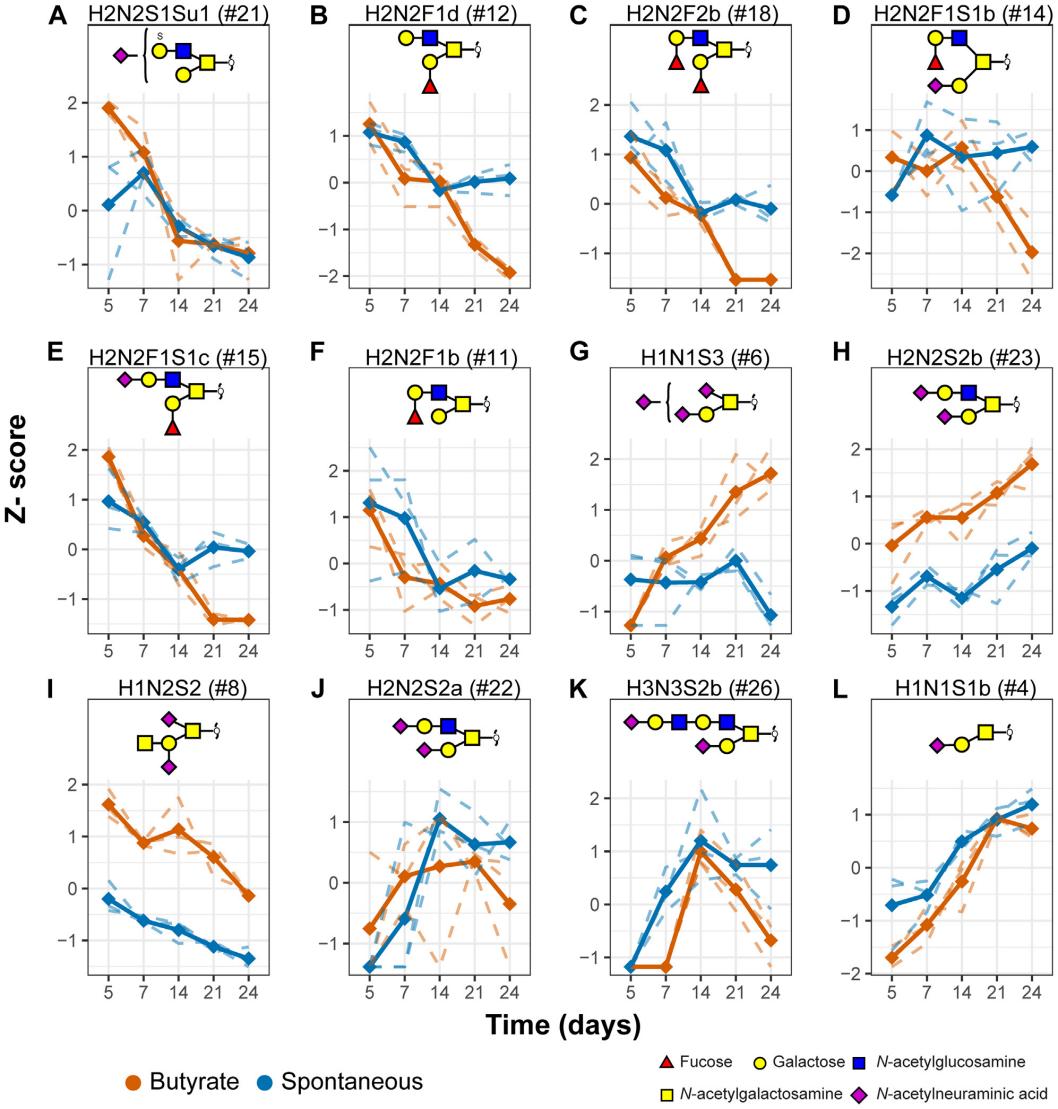
**Differentiation induces significant changes in *O*-glycan expression.** Differentiation induces significant downregulation (*A*–*F*; i) or upregulation (*G*, *H*, *J*, *K*, and *L*) of CaCo-2 *O*-glycans as well as some butyrate-specific changes. Spaghetti plots of the selected glycan abundances that show significant difference between time points selected from the ANOVA. The *dashed lines* represent the scaled z-scores of the measured values of each biological replicate, whereas the *continuous lines* represent the z-scores of the mean values per biological replicate. CaCo-2, *Ca*ncer *Co*li-2; Su, sulfation.

*O*-glycans that show a clear consecutive pattern with differentiation were selected for visualization (Fig. 3). Figure 3, *A*–*F* show the *O*-glycans that are significantly downregulated with differentiation and are carriers of terminal blood group antigens (H2N2F1b, H2N2F1d, H2N2F2b, H2N2F1S1b, and H2N2F1S1c) as well as sulfation (H2N2S1Su1). All *O*-glycans that increased overtime (Fig. 3, *G*, *H*, *J*–*L*) carried terminal *N*-acetylneuraminic acids and no fucosylation (H1N1S3, H2N2S2b, H2N2S2a, H3N3S2b, and H1N1S1b). The glycan with a disialyl motif, with composition H1N1S3 (Fig. 3*G*), showed an increase with time only in the butyrate-stimulated cells. Notably, the Cad antigen (GalNAcβ1-4(Neu5Acα2-3)Galβ1-3[Neu5Acα2-6]GalNAc) present on *O*-glycan with composition H1N2S2 (Fig. 3*I*) showed a downregulation with differentiation. However, it is significantly higher in the butyrate-stimulated group compared with the spontaneous differentiation group. Overall, we observed a downregulation of blood group antigen H fucosylation and upregulation of terminal sialylation with differentiation.

### Structural Identification of Glycan Species

Because of reports from the literature that provide contradictory *O*-glycan assignments, we made additional efforts to support the assignment of the specific *O*-glycan structures. As the *O*-glycomic profile of BSM has been very well characterized by both NMR and tandem MS, we used this as a reference standard (39, 40, 41). At *m/z* 895.34, two isomers with composition H2N2F1 were observed in our CaCo-2 samples (Supplementary Information 1, supplemental Fig. S4*A*; isomer *1* and *2a*). The major isomer expressed in CaCo-2 cell line (2a) matched with the major isomer in BSM standard (Supplementary Information 1, supplemental Fig. S4*B*; *2b*) by retention time and MS/MS spectrum, which allowed us to confirm that the major *O*-glycan isomer (*2a*) in the CaCo-2 samples carried the terminal blood group H antigen (40). The MS/MS spectra of this isomer (Supplementary Information 1, supplemental Fig. S4, *D* and *E*) show the presence of a highly abundant Z-ion (*m/z* 569.20 [H1N2]) indicating the occupancy of the 6′-antenna of the core 2 *O*-glycan. On the other hand, isomer *1* was found solely in CaCo-2 cells, and the MS/MS spectra (Supplementary Information 1, supplemental Fig. S4*C*) revealed the presence of terminal fucose linked to a type 2 *N*-acetyllactosamine on the 6′ antenna, indicated by the characteristic cross-ring fragment of the β1–4-galactosylated GlcNAc (*m/z* 409.02). Previous studies have reported an α1–2-linked terminal l-fucose, as part of blood group antigen H (42). In contrast, a recent study has postulated the presence of an unconventional terminal α1–6-linked fucose, yet evidence that would support this claim is lacking (30). In addition, two isomers with composition H2N2F2 at *m/z* 1041.40 were observed in our CaCo-2 samples (Supplementary Information 1, supplemental Fig. S5*A*, isomer *1* and *2a*). Isomer *2a* matched by retention time and MS/MS spectra with the major isomer of the BSM standard (Supplementary Information 1, supplemental Fig. S5, *B* and *D*; isomer *2b*), indicating a blood group antigen H–related terminal α1–2-fucose (30). Isomer *1* could not be identified by MS/MS and did not provide insightful differences in the fragmentation patterns because of low abundance. Interestingly, a recent study postulated the presence of two α1–6-linked terminal fucosides carried by a major isomer with composition H2N2F2 in CaCo-2, yet without providing sufficient experimental evidence (30). The structural identification of an *O*-glycan with composition H1N1F1S1 at *m/z* 821.30 is illustrated in Supplementary Information 1, supplemental Fig. S6. Based upon fragments *m/z* 530.23 and 512.14, this *O*-glycan carries a blood group antigen type 3. The signal at *m/z* 495.25 indicated an α2–6-linked sialic acid on the innermost GalNAc, which is in contrast to a previously reported annotation where the fucose was stated to be linked to the innermost GalNAc, whereas the sialic acid was linked to terminal galactose in α2–6 linkage (30). Structural elucidation of the *O*-glycan with composition H1N1S3 at *m/z* 1257.44 is shown in Supporting Information 1, supplemental Fig. S7. Two sialic acids appeared to be linked to each other, indicated by the presence of a characteristic fragment ion at *m/z* 581.18. While our data did not provide sufficient evidence to deduce the location of this disialyl motif, it has been previously described that this occurs on the α2–6-linked sialic acid linked to the innermost GalNAc (43). Moreover, structural elucidation of an *O*-glycan with Cad antigen, upregulated in the butyrate-stimulated cells, was depicted in Supplementary Information 1, supplemental Fig. S8. Overall, we observed a downregulation of terminal blood group H antigen expression and an upregulation of both galactose α2–3-sialylation and core GalNAc α2–6-sialylation with differentiation.

### Proteomic Analysis

Butyrate has been extensively used for stimulation of differentiation. However, to our knowledge, a quantitative proteomics approach has not been employed to study the effect as well as differences between spontaneous differentiation and butyrate-stimulated differentiation in the CaCo-2 cell line. Therefore, we performed quantitative bottom–up proteomics on the very same set of samples analyzed for the profiling of the *O*-glycome. The samples were digested with trypsin, isotopically labeled with a TMT, and analyzed by LC–MS/MS. With this approach, a total of 5050 proteins could be confidently quantified (mascot score >21, unique peptides ≥1; Supplementary Information 3, supplemental Table S2).

**Fig. 4:**
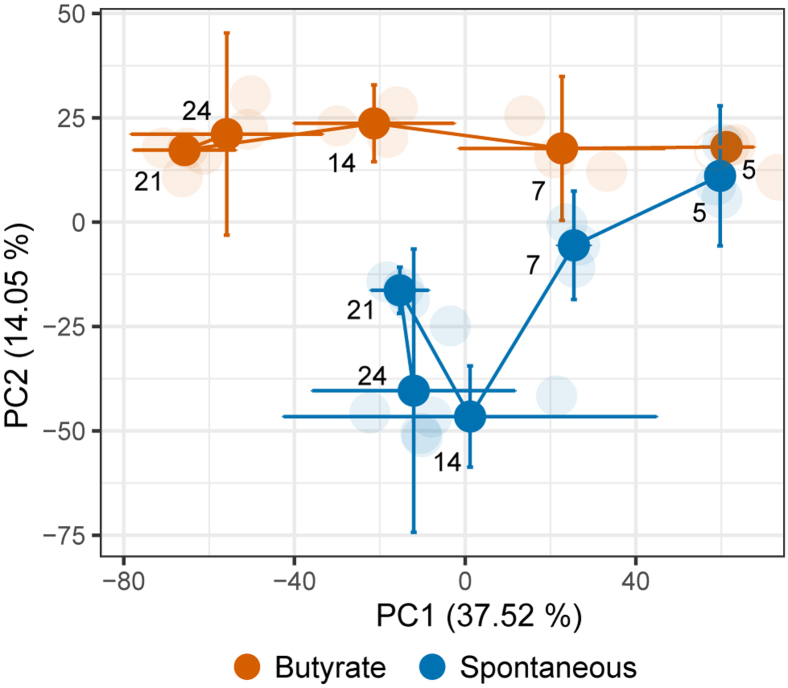
**The geometric trajectory visualization of proteomic changes with differentiation.** PCA model based on abundance of different proteins shows a separation between the butyrate-stimulated group (*orange*) and spontaneous differentiation (*blue*). The separation between different time points in the two groups is illustrated as a trajectory. The top two principal components (PCs) explain 51.57% of the variation within the data. The PCA scores from different biological replicates (*faded color*) were averaged to create the trajectory. PCA, principal component analysis.

To explore specific proteome variations that correlate to the differentiation of butyrate-stimulated samples, PCA was used (Supplementary Information 1, supplemental Fig. S9). The geometric trajectory in Figure 4 (based on the PCA model) illustrates changes in the cell proteome with differentiation as well as the differences in butyrate-stimulated cells. At day 5, both groups cluster closely together, whereas the changes start to be more prominent at day 7 culminating at day 14. In the nonstimulated cells, the proteome appears to stabilize after day 14. In contrast, butyrate stimulation induced significant changes in the proteome until day 21. Proteins that show statistically significant changes (two-way ANOVA, Bonferroni corrected *p* value <0.05) with time, with butyrate stimulation, as well as with both time and with butyrate stimulation are listed in Supplementary Information 3, supplemental Tables S4–S6, respectively. Gene Ontology (GO) enrichment analysis of those proteins revealed changes in proteins involved in spliceosome, ribosome, amino acid degradation, fatty acid metabolism including tricarboxylic acid cycle and pyruvate metabolism (Supporting Information 1, supplemental Fig. S10). Interestingly, significant upregulation of placental type and germ cell type APs (ALPP and ALPG) and cytokeratin 20 (KRT20) were found, but only a trend toward upregulation for the intestinal AP (ALPI) was found in the butyrate-stimulated cells (Supplementary Information 1, supplemental Fig. S11). Specific transcription factors known for their role in regulation of colon differentiation also showed a change (Supplementary Information 1, supplemental Fig. S12). Hepatocyte nuclear factor 1α (HNF1A) decreased in abundance over time in both groups, and in the butyrate-stimulated cells, it continued to decline after 14 days. Hepatocyte nuclear factor 4α (HNF4A) showed an upregulation after 5 days, with a decline after 7 days in spontaneous differentiation. Whereas in the butyrate-stimulated cells, it continued to rise until 14 days, followed by a decline. Transcription factors GATA6 and FOXA1 showed a continuous decrease in abundance both in spontaneous and butyrate-stimulated cells. Although not statistically significant, transcription factor CDX2 decreased over time in both groups. All transcription factors that show a change with differentiation are listed in Supplementary Information 3, supplemental Table S9. The majority of the proteins changing specifically with butyrate stimulation are related to cell metabolism (hsa01100-Kegg pathways).

In order to relate the *O*-glycomic changes to changes in the abundance of proteins involved in the glycan or monosaccharide precursor biosynthesis, as well as *O*-glycoproteins, we continued our analysis of the proteomic data by selecting the proteins involved in the GO ontologies-glycosylation, GO-monosaccharide transport, as well as proteins reported to be *O*-glycosylated in different cell lines discovered by the Simple cell approach (44). From this selection, 120 proteins showed a statistically significant change with time, whereas only 36 proteins showed a significant change with both time and butyrate stimulation (Supplementary Information 3, supplemental Tables S7 and S8, respectively). Interestingly, the only mucin that showed an upregulation over time was MUC13 (Supplementary Information 3, supplemental Table S7). In addition, changes were detected in monosaccharide transport–related protein including solute carrier family facilitated monosaccharide transporters (SLC2A1, SLC2A3, and SLC2A5) as shown in Supplementary Information 1, supplemental Fig. S13. Also, the abundances of polypeptide GalNAc transferases 2 and 3 (GALNT2, GALNT3, respectively) were increased over time (Supplementary Information 1, supplemental Fig. S13). However, other glycosyltransferases involved in the biosynthesis of *O*-glycans did not show a significant change or were not detected with our proteomics workflow. Interestingly, the abundance of GDP-mannose 4,6-dehydratase (GMDS) was downregulated with differentiation. However, the change was not statistically significant.

**Fig. 5:**
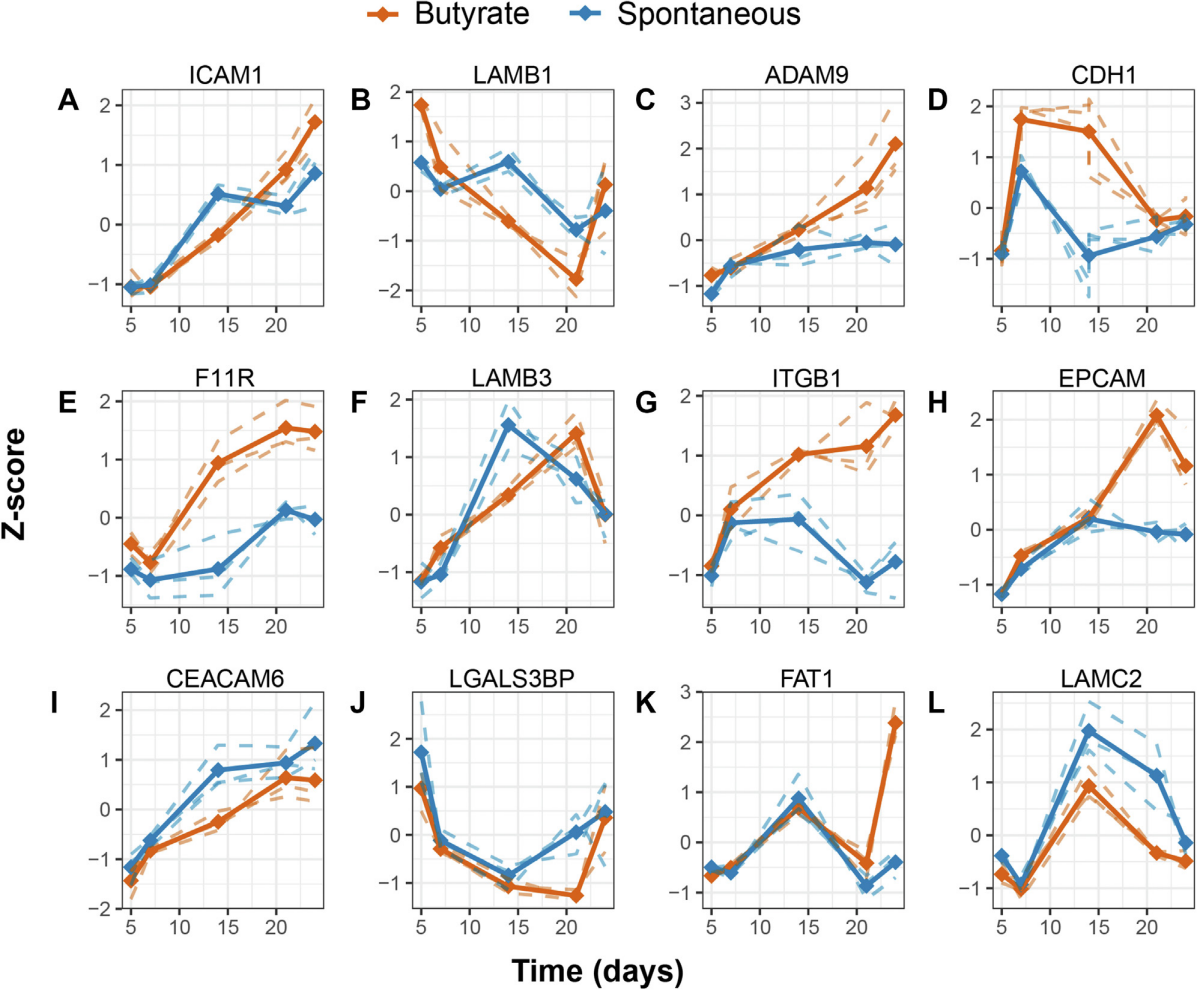
**Differentiation induces significant changes in the abundance of cell adhesion *O*-glycoproteins.** Spaghetti plots of the changes in abundances of proteins involved in cell adhesion that show significant difference between groups selected from the ANOVA (*A–L*). The *dashed lines* represent the scaled Z-scores of the measured values of each biological replicate, whereas the *continuous lines* represent the Z-scores of the mean values per biological replicate.

Significant changes with differentiation were observed for the abundances of different *O*-glycoproteins involved in cell adhesion such as intercellular adhesion molecule 1 (Fig. 5*A*), laminin subunit beta 1 (LAMB1; Fig. 5*B*), disintegrin and metalloproteinase domain–containing protein 9 (ADAM9; Fig. 5*C*), E-cadherin (Fig. 5*D*), junctional adhesion molecule 1 (F11R; Fig. 5*E*), laminin subunit beta-3 (Fig. 5*F*), integrin beta 1 (ITGB1; Fig. 5*G*), epithelial cell adhesion molecule (EPCAM; Fig. 5*H*), carcinoembryonic antigen–related cell adhesion molecule 6 (CEACAM6; Fig. 5*I*), galectin 3-binding protein (LGALS3BP; Fig. 5*J*), protocadherin fat 1 (FAT1; Fig. 5*K*) and laminin subunit gamma 2 (Fig. 5*L*). Interestingly, butyrate-stimulated cells showed higher abundances of cell adhesion proteins such as protocadherin (FAT1), E-cadherin (CDH1), and epithelial cell adhesion molecule than spontaneously differentiated cells (Fig. 5 and Supplementary Information 3, supplemental Table S8).

### Data Integration

**Fig. 6:**
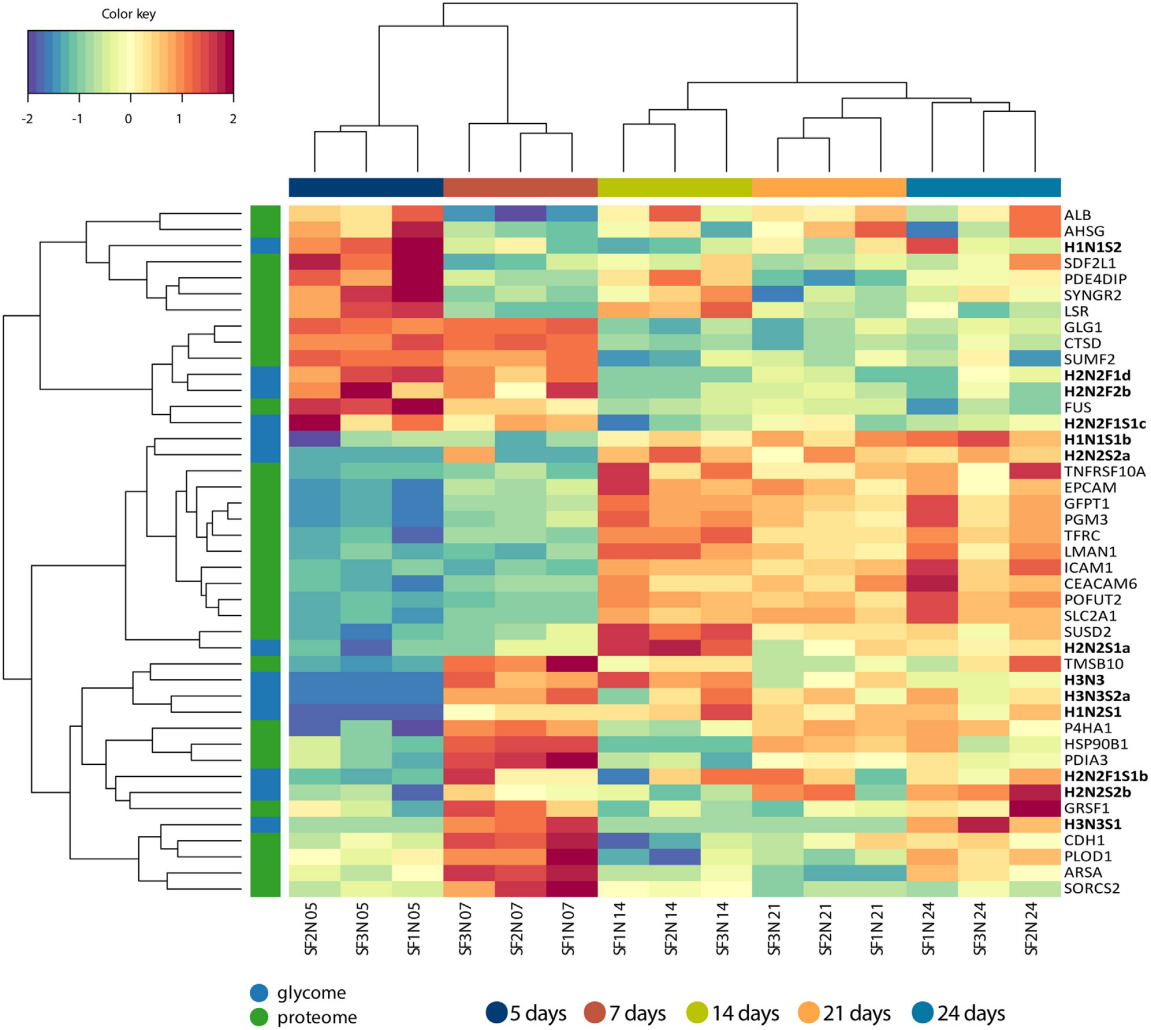
**Multiomics signature of spontaneous differentiation.** Clustered image map illustrating changes in both proteome and glycome with differentiation based on a combination of the partial least square (PLS) regression generalized for the multiple matched datasets and LASSO (least absolute shrinkage and selection operator)–based variable selection. The model was tuned for the maximal correlation.

**Fig. 7:**
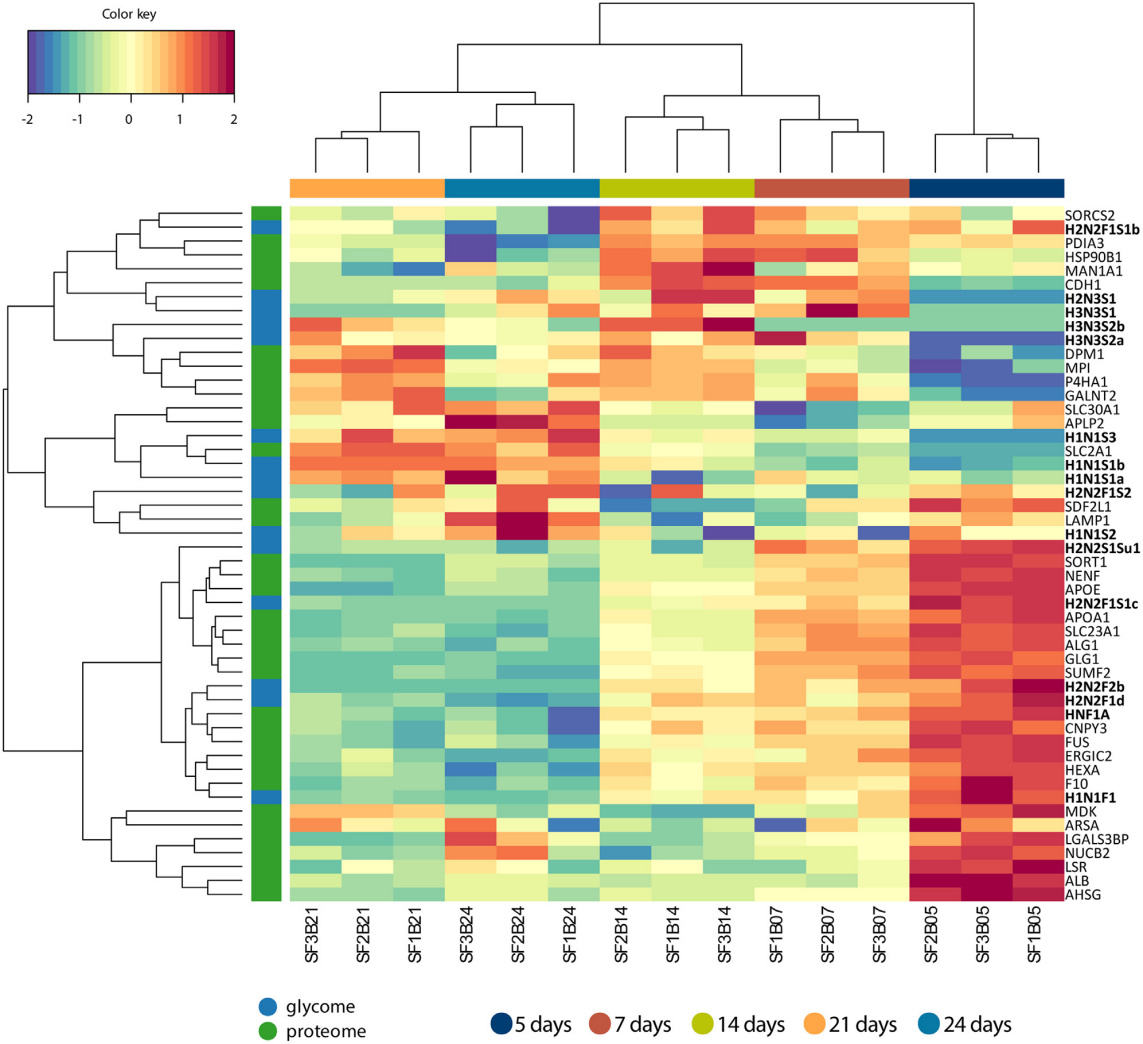
**Multiomics signature of butyrate-stimulated differentiation.** Clustered image map illustrating changes in both proteome and glycome with differentiation based on a combination of the partial least square (PLS) regression generalized for the multiple matched datasets and LASSO (least absolute shrinkage and selection operator)–based variable selection. The model was tuned for the maximal correlation.

To identify associations between the proteomic and *O*-glycomic changes with differentiation, we used a data integration approach (MixOmics) (45). For this purpose, only the aforementioned proteins of interest were included in the analysis. Because of the complexity of the model, the proteome and *O*-glycome differentiation signatures over time were integrated separately for the spontaneous and butyrate-stimulated cells. The clustered image map indicated that changes on the *O*-glycome level highly associated with the changes on the proteome level in spontaneous differentiation, revealing relationships with specific enzymes and monosaccharide transporters (Fig. 6). Among others, an upregulation of SLC2A1, a monosaccharide transporter of a wide range of aldoses, is seen at this stage of differentiation, as well as glutamine-fructose-6-phosphate aminotransferase 1 (GFPT1) and phosphoacetylglucosamine mutase (PGM3) which catalyzes the conversion of GlcNAc-6-P into GlcNAc-1-P during the synthesis of UDP-GlcNAc. The changes in the later time points are very similar as for day 14 due to the stabilization that was observed in the proteome and *O*-glycome of spontaneously differentiated cells. On the other hand, butyrate-stimulated differentiation induced significant changes on the proteome level even until day 21, and less prominent changes until day 24 (Fig. 7). Downregulation of, among others, apolipoproteins E (APOE) and A1 (APOA1), alpha-2-HS-glycoprotein (AHSG), albumin (ALB), lipolysis-stimulated lipoprotein receptor (LSR), sodium/ascorbate transporter (SLC23A1), chitobiosyldiphosphodolichol beta-mannosyltransferase (ALG1), beta-hexosaminidase (HEXA) was observed in spontaneously differentiated cells. On the other hand, butyrate-stimulated differentiation induced subunit alpha (EXA) and hepatocyte nuclear factor 1-alpha (HNF1A) downregulation, which were correlated with fucosylated *O*-glycomic signatures, such as H2N2F1S1c, H2N2F2b, H2N2F1d, and H1N1F1. Furthermore, it also induced downregulation of galectin-3-binding protein (LGALS3BP). Moreover, butyrate stimulation induced upregulation of proteins involved in the monosaccharide metabolism, such as mannose-6-phosphate isomerase (MPI) involved in the synthesis of the GDP-mannose as well as solute carrier family 2, facilitated glucose transporter member 1 (SLC2A1). Upregulation of proteins involved in the biosynthesis of *N*- and *O*-glycans was also induced by butyrate stimulation such as mannosyl-oligosaccharide 1,2-alpha-mannosidase IA (MAN1A1) and dolichol-phosphate mannosyltransferase subunit 1 (DPM1). Upregulation of polypeptide GALNT2 showed a similar pattern to the upregulation of the following sialylated *O*-glycans H1N1S3, H1N1S1b, H1N1S1a, H2N2F1S2, and H1N1S2 all showing increasing abundances with cell differentiation.

The associations found with HNF1A triggered us to explore if other transcription factors associated with the *O*-glycomic changes. Therefore, we performed the integrative analysis selecting only the *O*-glycans and the transcription factors that showed a significant change with differentiation (Supplementary Information 3, supplemental Table S9). Downregulation of fucosylation in spontaneously differentiatiated cells showed associations with FOXA1, FOXP4, GATA6, STAT1, and HNF1A transcription factors. Specifically, HNF4A expression was associated more with the expression of H2N2S1Su1 and H3N3S1 glycomic signatures (Supplementary Information 1, supplemental Fig. S14). Similar to HNF1A, which showed associations with glycomic signatures carrying terminal blood group H (H2N2F2b and H2N2F1S1c) (Fig. 7), transcription factor HNF4A associated with H2N2F1S1b in the butyrate-stimulated cells (Supplementary Information 1, supplemental Fig. S15). On the other hand, upregulation of sialylated species associated with NF-kappa-B transcription factor P65 (RELA) and MYC promoter–binding protein 1 (ENO1) in butyrate-stimulated cells.

## Discussion

In this study, we observed changes in the CaCo-2 *O*-glycome that correlated to differentiation over time. Both butyrate-stimulated and spontaneously differentiated cells were accompanied by the elevation of galactose α2–3-sialylation and a downregulation of the terminal blood group H antigen over time. However, it should be noted that there are contradictory reports in the literature regarding the correlation of the terminal blood group H-antigen expression with differentiation. Namely, Murakami *et al.* reported no detection of the H-type blood group antigen, whereas Vincente *et al.* reported diffused expression of H-type antigen, Amano *et al.* reported higher H-type 1 antigen expression with differentiation and Xu *et al.* reported no changes in the *O*-linked fucosylation (30, 46, 47, 48). Moreover, an increase in fucosylated *N*-glycans and a concurrent decrease of high mannose type glycans was previously reported for spontaneous differentiation of CaCo-2 cells (49). It is important to note that there were considerable differences in the experimental design of these studies regarding induction of differentiation, days in culture, as well as detection methods. Moreover, it was previously postulated that CaCo-2 cells can form different subpopulations during growth and differentiation, and considerable differences between laboratories have previously been reported for identical experimental conditions (50, 51). Therefore, we first validated that the cells showed changes in morphology (Fig. 1*B*) related to dome formation observed previously with cell differentiation, associated with unidirectional flux of ions and water through the polarized monolayer (37, 38). Moreover, we monitored the changes in the activity of AP (Fig. 1*A*), which is a well-established marker of epithelial differentiation. However, it must be noted that because of cellular heterogeneity the differentiation process occurs in a mosaic pattern, with some areas expressing fully differentiated cells, whereas other areas contain less differentiated cells (50). This is why we compared three biological replicates of cells, subcultured from the same batch within identical time frames and culture conditions. Thereafter, we aimed at deciphering differences between spontaneous and butyrate-stimulated changes with cell differentiation focusing on the *O-*glycome and proteome by MS.

Unlike differentiated enterocytes, cancer cells produce energy predominantly through anaerobic glycolysis in the cytosol and not through the citric acid cycle and oxidative phosphorylation of fatty acids. This phenomenon is referred to as the Warburg effect. The butyrate is then accumulated in the cells and acts as a histone deacetylase inhibitor resulting in hyperacetylation and transcriptional activation, leading to inhibition of cell proliferation and cell differentiation (52). However, previous metabolomic analysis demonstrated a shift from glycolysis to oxidative phosphorylation occurring upon butyrate treatment of colon cancer cells, resulting in metabolic reprogramming (53). Similarly, our GO enrichment analysis of the proteins significantly changing with differentiation revealed extensive changes in abundances of proteins involved in fatty acid metabolism (Supporting Information 1, supplemental Fig. S10). Thus, effects of butyrate on cell cycle arrest, AP activity, and apoptosis are likely not present in the differentiated CaCo-2 cells, presumably caused by rapid use of butyrate as energy source (54).

Our study, on the relative abundances of individual *O*-glycans, showed that the CaCo-2 *O*-glycome for spontaneous differentiation stabilizes after day 14, which is in line with a proteomics analysis on the spontaneous differentiation of CaCo-2 cells previously published by Stierum *et al.* (25). The *O*-glycome for the butyrate-stimulated group behaved significantly differently and did not stabilize after day 14. Noticeable, in the spontaneously differentiated cells, the expression of terminal blood group antigens carrying an α-1,2-fucoside stabilized at day 14, whereas most of these α-1,2-fucoside-carrying antigens in the butyrate-stimulated cells continued decreasing after day 14. Fucoside moieties on glycans are increasingly recognized as critical attributes for cell–cell interactions and signaling processes (53). The decrease of type 2 blood group H fucosylated glycans with a subsequent increase in type 1 blood group H and Lewis B (Le^B^) glycans was previously correlated to the spontaneous differentiation of CaCo-2 cells (48, 55). This effect was linked to the changes in the activity of specific enzymes responsible for type 1 blood group H biosynthesis such as β1–3-galactosyltransferase and α1–2-fucosyltransferase (56). We did not find the blood group H type 1 antigens on *O*-glycans, which might indicate that other types of glycans are carriers of the antigen such as glycosphingolipids or *N*-glycans. For further studies, it would be interesting to investigate butyrate-induced fucose downregulation for different glycan types together with its implications on cell properties. At the same time, upregulation of sialylated glycan species is induced upon differentiation, which was previously correlated to differentiation assessing the expression of corresponding glycosyltransferases such as ST3GAL6 (57, 58, 59).

The core 1 *O*-glycan carrying a Cad antigen showed a downregulation over time for both groups, correlating to differentiation, but a significantly higher expression was observed for the butyrate-stimulated cells compared with the spontaneously differentiated cells. Structures with terminal Cad and Sda epitopes have previously been described as characteristic for normal colon tissue (60), mainly carried by core 3 glycans (61). However, in line with our findings, most studies report a downregulation of the Cad antigen with differentiation in CRC cell lines (32, 60). β1–4-*N*-acetylgalactosaminyl transferase 2 (B4GALNT2) is responsible for the addition of the terminal GalNAc residue in a β1–4 linkage to the sialylated galactose. It has previously been observed that the expression of the Cad antigen and the *B4GALNT2* gene is regulated by promoter DNA methylation (62). Recent literature reported the capability of butyrate to trigger DNA demethylation in CRC cell lines (63). Promoter demethylation triggered by butyrate, followed by differential expression of the *B4GALNT2* gene and corresponding Cad antigen, could explain the significantly higher expression of the Cad antigen in the butyrate-stimulated group compared with the spontaneous differentiation group.

Another interesting *O*-glycan that shows an upregulation in the butyrate-stimulated samples compared with the spontaneously differentiated samples (after day 14) is the *O*-glycan with composition H1N1S3 (Fig. 3*G*). Tandem MS revealed that this *O*-glycan carries a disialic acid unit (Supplementary Information, supplemental Fig. S7), potentially with an α2–8 glycosidic linkage (64). Previously, an upregulation of α2–8-sialyltransferase *ST8SIA6* mRNA expression was reported in CaCo-2 cells that underwent spontaneous differentiation (49). Details regarding the biological function of this disialic acid motif remain unknown, making it an interesting structure for future studies. Moreover, an increase in core 2 sialylated *O*-glycans was observed in differentiated cells, which is in line with previously reported changes in the expression of α2–3-sialyltransferases (ST3GAL4 and ST3GAL6) as well as core 2 synthases (GCNT4 and GCNT3) (49). In addition, the previously reported upregulation of β1,3-*N*-acetylglucosaminyltransferase 3 (49) could be responsible for the upregulation of the H3N3S2b glycan, by adding another LacNAc to the 6 arm of the *O*-glycan.

BSM and its *O*-glycan repertoire has been very well characterized by both tandem MS and NMR (40, 41) and served in our study as a valuable reference standard allowing us to assign specific glycan linkages. We were able to confirm that the dominant isomers of *O*-glycans with composition H2N2F2 and H2N2F1 are carrying α1–2 linked terminal fucoses (Supplementary Information, supplemental Figs. S4 and S5, respectively), whereas a recent study reported the presence of an unconventional terminal α1–6-linked fucose (30), yet without providing sufficient experimental evidence. However, our approach did not accommodate the assignment of all *O*-glycan isomer linkages, as several of them were not present in the BSM standard.

The stabilization of the *O*-glycome for spontaneously differentiated cells after day 14 is in line with the stabilization of the proteome as reported previously by Stierum *et al.* (25), 14 days postconfluence. In this study, we were able to study both the effect of butyrate stimulation and spontaneous differentiation on the same sample set used for profiling the *O*-glycome. In agreement with the dynamics of their *O-*glycomics data, the proteome of the butyrate-stimulated cells also showed further changes 14 days postconfluence, indicating an important role for butyrate-stimulated cellular regulation in the late phase of differentiation. In order to relate the previously mentioned *O*-glycomic changes to changes in the abundance of specific proteins, we continued our analysis of the proteomic data by selecting the proteins involved in the GO ontologies – Glycosylation, Monosaccharide transport, as well as *O*-glycoproteins (44).

An upregulation of specific monosaccharide transporters SLC2A1 (GLUT1), SLC2A3 (GLUT3) and SLC2A5 (GLUT5) was seen in butyrate-stimulated differentiated cells, important for uptake of glucose and other monosaccharides into the cell, which was previously associated with differentiated CaCo-2 cells (65, 66). Changes in the expression of enzymes involved in metabolic conversion of monosaccharide precursors were also observed with differentiation. Namely, hexokinase that converts glucose into glucose-6-P (HK2), glutamine-fructose-6-phosphate aminotransferase 1 (GFPT1) and phosphoacetylglucosamine mutase (PGM3), both involved in the biosynthesis of UDP-GlcNAc, are upregulated with differentiation. These enzymes are important rate-limiting enzymes of the hexosamine pathway, regulating the availability of monosaccharide precursors for *N*- and *O*-linked glycosylation of proteins. Importantly, mammalian cells can produce sialic acid precursors by conversion of GlcNAc from the hexosamine pathway to ManNAc and subsequently CMP-NeuAc (67). This pathway is different from the *de novo* biosynthesis of GDP-Man and GDP-Fuc. Interestingly, the only monosaccharide present in the cell culture media is glucose; therefore, the availability of other monosaccharide precursors for glycan biosynthesis is dependent on their intracellular *de novo* biosynthesis from glucose. The upregulation of the hexosamine pathway, and subsequent production of CMP-NeuAc, can explain the upregulation of sialylation and downregulation of fucosylation with differentiation. Previously, we compared the *O*-glycome of 26 CRC cell lines and revealed associations with differentiation where the well-differentiated cells expressed Lewis-type fucosylation, whereas the undifferentiated cells, such as the CaCo-2 cell line, showed expression of *O*-linked blood group H antigens (31). This could explain why there was not an increase in Lewis-type fucosylation observed in this study upon differentiation. However, it should also be taken into account that, although developed from colon carcinoma, CaCo-2 cells showed quite specific glycosylation, very rich in terminal blood group antigen H expression compared with other CRC cell lines (31). Moreover, they differentiate into small intestinal enterocyte-like cells, with a consequent loss of colonocyte properties (68).

Studies on a molecular level for glycosyltransferases are known to be challenging, as many of them are low abundance proteins (69). With our current method, we were able to detect an increase in abundance of polypeptide GalNAc transferases 2 and 3 with differentiation (GALNT2 and GALNT3, respectively), followed by a decline in the late stages of differentiation. Previously, knockout of GALNT2 and GALNT3 in human organotypic skin models caused impaired cell adhesion and decreased differentiation, respectively. This implies importance of these enzymes initiating *O*-GalNAc glycosylation for cell–extracellular matrix interactions and epithelial differentiation (70). In addition, proteins such as F11R, BCAM, ADAMTSL4, LGALS3BP, and laminin are found to be GALNT2-specific targets, whereas protocadherin (FAT2) and cathepsin (CTSD) are found to be GALNT3-specific targets in skin cells (70). Another study pinpointed apolipoprotein E, nucleobindin-2, lipolysis-stimulated lipoprotein receptor, laminin (LAMC2), coagulation factor X, and protein disulfide-isomerase A3 as GALNT3 and GALNT6 targets (71). These observations could provide interesting perspectives to study the GALNT-specific targets in the colon and how the glycosylation of the specific proteins influences their function in cell adhesion during differentiation.

Interestingly, we identified only four mucins in our study, of which only one (MUC13) showed a change with differentiation. The limited coverage of the mucin glycoproteins is likely attributed to the analysis of cell lysates rather than the secretomes, which most likely contain large amounts of secreted mucins. Moreover, low efficacy of trypsin for digestion of mucins, often lacking arginine/lysine residues in heavily glycosylated clustered regions, may contribute to the limited coverage (72). Although not many mucins were detected, a good coverage of other *O*-glycoproteins was achieved, of which 98 showed a statistically significant change with time, and 29 with butyrate stimulation.

To identify associations between proteomics and glycomics changes with differentiation, we continued using a data integration approach (MixOmics) (45). The specific butyrate pattern showed higher abundances of cell adhesion proteins in later stages of differentiation. Similarly, changes of cell adhesion and cell junction proteins were observed before for CaCo-2 cells undergoing spontaneous differentiation (49). Those proteins show a similar change in abundance as sialylated *O*-glycan species (Fig. 6). Changes in the abundances of specific *O*-glycoproteins could contribute to the changes in the *O*-glycome. However, a direct link between specific *O*-glycans and *O*-glycoproteins could not be established without performing *O-*glycoproteomic experiments. The *O*-glycoproteins included in our assessment have been identified by Steentoft *et al.* (44) by mapping of *O*-glycosites from genetically engineered Simple Cells. A total of 662 O-glycoproteins have been identified in this study. Site occupation of these proteins has been demonstrated by MS upon blocking the *O*-glycome extension pathways resulting in glycoproteins with a single GalNAc on each site. While we report associations between *O*-glycome and inferred *O*-glycoproteome in our study, glycopeptide-based *O*-glycoproteomic studies are needed to obtain insights into *O*-glycoproteomic changes. Such studies suffer from poor glycopeptide ionization within complex peptide mixtures, even upon enrichment with hydrophilic interaction liquid chromatography and lectin affinity chromatography (73). Moreover, fragmentation techniques need to be specifically optimized to deliver enough informative fragments for both the glycan and peptide portion, which can be challenging for a wide range of peptides from complex mixtures (74). Unfortunately, because of these limitations, we could not directly link the changes in the *O*-glycome with specific *O*-glycoproteins.

Future studies using affinity purification selecting for specific proteins or specific glycosylation by lectins could potentially reveal if the associations are real. Previously, a genetic engineering approach that limits the glycan diversity to a single GalNAc or sialyl-GalNAc enabled enrichment of cell *O*-glycoproteome by lectin affinity chromatography and revealed the complexity of the cell *O*-glycoproteome (75). Although this approach is invaluable for the discovery of site occupancy, it does not give information about the glycan microheterogeneity on the site. While, specific *O*-glycosylation of cell adhesion *O*-glycoproteins may play an important role in cell differentiation, the structure–function relationship of glycosylation in these proteins involved in adhesion is still to be deciphered.

Remarkably, we observed a general downregulation of abundances of transcription factors involved in epithelial differentiation such as HNF1A, GATA6, and FOXA (76). Whereas, abundance of HNF4A, the key regulator of the expression of intestinal genes in CaCo-2 model (76), increased by day 14 in butyrate-stimulated cells, in line with previous research where an upregulation on mRNA and protein levels was observed (77). Moreover, it was previously shown that HNF4A also regulates the HNF1A and CDX2 promoter activity (77) and the functional interaction between HNF1A and CDX2 was demonstrated before in CaCo-2 cells (78). Although no unique peptides for CDX1 were identified in our study, the previously described target protein cytokeratin 20 showed upregulation with differentiation (79). The activity of these transcription factors depends on a complex network of interactions and not solely on the abundance of the protein product. The integrative analysis with transcription factors and *O*-glycans (Supplementary Information 1, supplemental Figs. S14 and S15) reveals new potential regulators of glycosylation, such as FOXA1, FOXP4, and STAT1. However, further studies are required to validate these hypotheses.

Interestingly, in the butyrate-stimulated differentiation, the downregulation of HNF1A transcription factor correlated with fucosylated glycomics signatures, such as H2N2F1S1c, H2N2F2b, H2N2F1d, and H1N1F1 (Fig. 7). HNF1A is a transcription factor expressed in several organs including the intestine and stomach. Genome-wide association studies by Lauc *et al.* (80) identified HNF1A as a key regulator of fucosylation. It was demonstrated that HNF1A activates antennary FUTs (FUT3–11 in liver cell lines) and downregulated core FUT8. In addition, it showed that HNF1A upregulated GMDS, an important enzyme in *de novo* d-fucose biosynthesis pathway, which increases the availability of GDP-fucose precursor for fucosylation of glycans (80). While a trend in downregulation of GMDS was seen on the proteome level, it did not meet the significance threshold. Nevertheless, our data indicate that there might be an important role of HFN1A as a master transcriptional regulator of fucosylation in CaCo-2 cells, where downregulation of HNF1A correlates with the downregulation of fucosylation upon differentiation. HNF1A downregulation can lead to the limitation of d-fucose availability and consequently upregulation of terminal sialylation, which is in direct competition with terminal blood group H antigen biosynthesis. Unfortunately, we did not detect the CDX1 transcription factor in our proteomics study, a previously reported transcriptional regulator of Lewis-type fucosylation on *N*-glycans (32, 81) and the detected CDX2 showed no statistically significant change. Moreover, the CaCo-2 cell line, among other undifferentiated cell lines, previously showed a predominant expression of *O*-linked blood group H fucosylation, and associations could be seen with HNF4A transcription factor (31). This further supports the hypothesis that HNF1A and HNF4A might be the key regulators of *O*-linked glycan fucosylation in CaCo-2 cells during cell differentiation. Several other associations between specific *O*-glycan structures and protein expression are reported in this study and could potentially be the start of new endeavors, which may have important implications for understanding colon cancer (de-)differentiation (80).

In this study, an integrative approach was used to establish relationships between changes in cell *O*-glycome and proteome that occur with cell differentiation. However, to further unravel the interplay between *O*-glycomic and proteomic changes, *O*-glycoproteomic analyses are needed, which still remains a challenging task for complex biological samples. Moreover, functional studies can be employed to validate the suggested transcriptional regulators of glycosylation and their role in cell differentiation. Which, eventually, will provide insights about the role of glycosylation cell adhesion and the role of fucosylation and sialylation in cell differentiation.

## Conclusions

Differentiated CaCo-2 cells are an important and widely used model cell line. However, the glycome expression might change dependent on the growth and bacterial metabolites available during the culturing process. In this study, an untargeted in-depth screening of the CaCo-2 cell line was performed, identifying specific *O*-glycans that mark butyrate-induced epithelial differentiation. Changes in the cell proteome were also investigated, leading to important insights into the changes in cell adhesion proteins, as well as the potential influence of *de novo* monosaccharide precursor biosynthesis on the *O*-glycome profile changes with differentiation. Considering the only monosaccharide supplied in cell culture media is glucose, this has important implications for future studies investigating colonocyte differentiation in culture. While interesting structures were identified, further studies are required to identify their role in maintaining homeostasis in the epithelium. In the future, this might allow us to gain a better understanding of the constantly changing *O*-glycome at the gut–microbiota interface as correlated to bacterial hydrolase activities and its relevance to maintaining homeostasis or role in dysbiosis.

## Data Availability

The raw mass spectrometric glycomic data files that support the findings of this study are available in GlycoPOST (82) repository. The following are the supplemental data related to this article: in mzXML format, with the identifier GPST000256.0, accessible *via* the following link https://glycopost.glycosmos.org/preview/1167164470620fc439c3cc9 (access code: 7405). The MS proteomics data have been deposited to the ProteomeXchange Consortium *via* the PRIDE (83) partner repository with the dataset identifier PXD037450. Annotated MS/MS spectra are available *via* the Unicarb DR repository.

## Supplemental data

This article contains supplemental data (39, 40, 84).

## Conflict of interest

The authors declare no competing interests.
