# Supplementary material for: O-Glycomic and Proteomic Signatures of Spontaneous and Butyrate-Stimulated Colorectal Cancer Cell Line Differentiation
Source: Mol Cell Proteomics. 2023 Jan 18;22(3):100501. doi: 10.1016/j.mcpro.2023.100501 (PMC9999233; doi:10.1016/j.mcpro.2023.100501)
Supplement: Supporting Information 1 [file mmc1.docx]

**Supporting Information I**

*O*-glycomic and proteomic signatures of spontaneous and butyrate-stimulated colorectal cancer cell line differentiation

K. Madunić^a^, Y.M.C.A. Luijkx^b,c^, O.A. Mayboroda^a^ , G.M.C. Janssen^a^, P.A. van Veelen^a^, K. Strijbis^c^,T. Wennekes^b^, G.S.M. Lageveen-Kammeijer^a^, M. Wuhrer^a,^*

^a^ Center for Proteomics and Metabolomics, Leiden University, The Netherlands.

^b^ Department Chemical Biology and Drug Discovery, Utrecht Institute for Pharmaceutical Sciences and Bijvoet Center for Biomolecular Research, Utrecht University, Utrecht, The Netherlands.

^c^ Department Biomolecular Health Sciences, Utrecht University, Utrecht, The Netherlands.

* Correspondence and requests for materials should be addressed to M. Wuhrer (email: m.wuhrer@lumc.nl)

**Supplemental Figure S1. Robustness of the glycomic workflow a)** Variability of the sample preparation workflow for three technical replicates from the same cell lysate (five days – spontaneous differentiation) **b)** Variability of five measurements of bovine submaxillary mucin released *O*-glycans across five  days.  H: hexose; N: *N*-acetylhexosamine; F: deoxyhexose; S:*N*-acetylneuraminic acid; Sg:*N*-glycolylneuraminic acid. The *O*-glycan isomers are labeled by a, b or c after the composition.


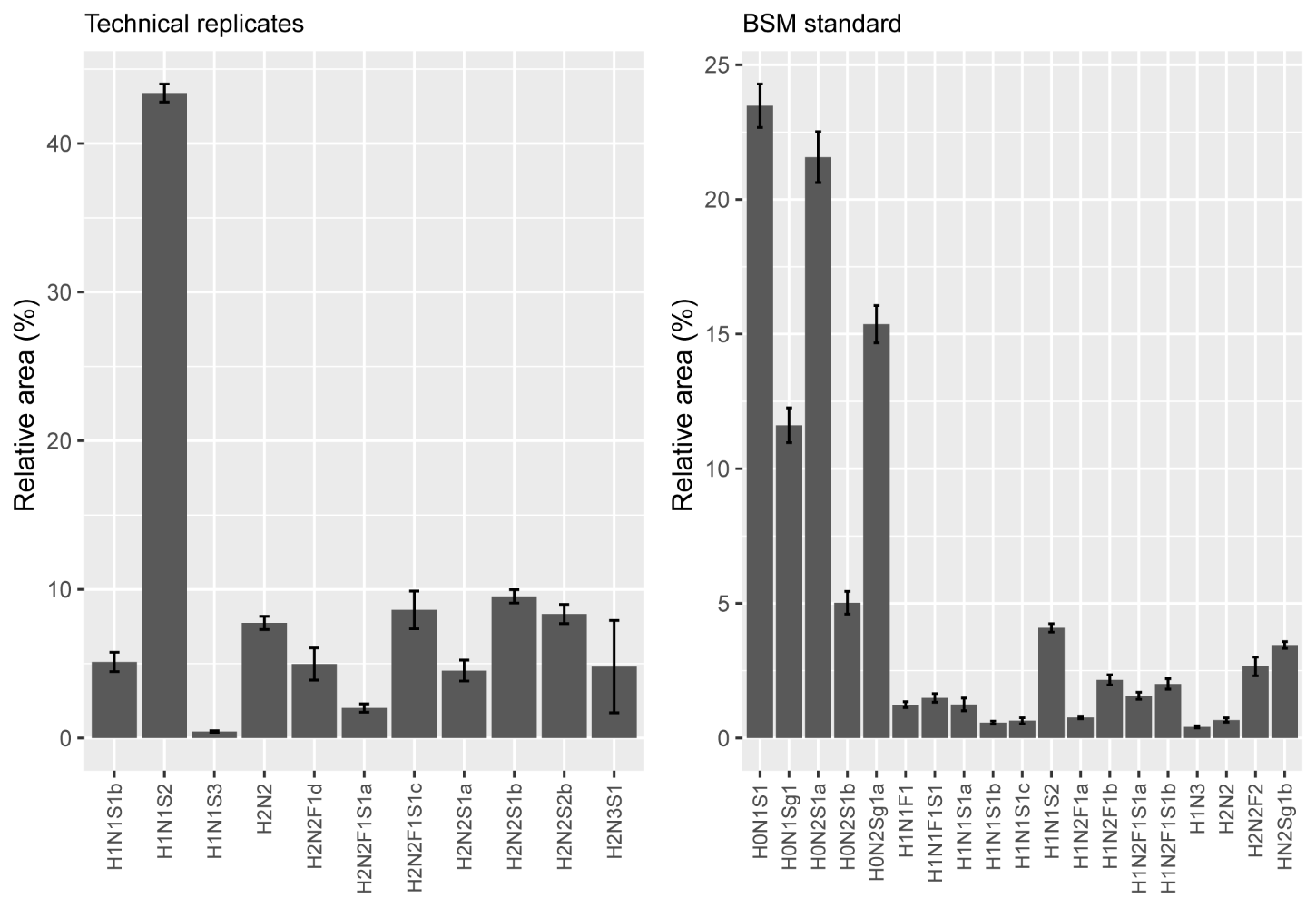


**A**

**B**

**Supplemental Figure S2. PCA model based on relative abundances (%) of released *O*-glycans. a)** PCA scores plot with three biological replicates (labeled 1, 2 or 3) per condition (NaBu/spontaneous) and per timepoint in days (5D, 7D, 14D, 21D, and 24D). The top two principal components (PC) explain 55,1 % of the variation within the data. **b)** The PCA loadings plot displays the variables (*O*-glycans) that drive the separation in the PCA model.


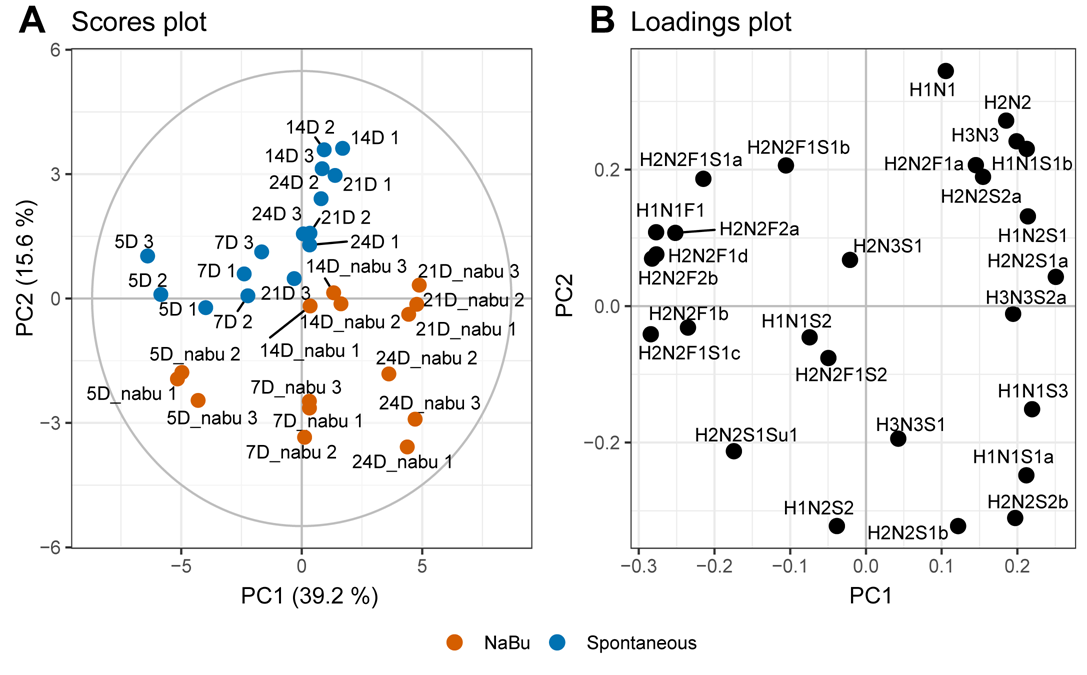


**Supplemental Figure S3. Relative abundance of *O*-glycans that show a significant difference with differentiation.** Geometrical tile of the scaled relative abundances (%) of individual *O*-glycans (y-axis) across different timepoints (x-axis) selected from the analysis of variance (ANOVA) with NaBu stimulation **(a)** and without (spontaneous differentiation) **(b)**.


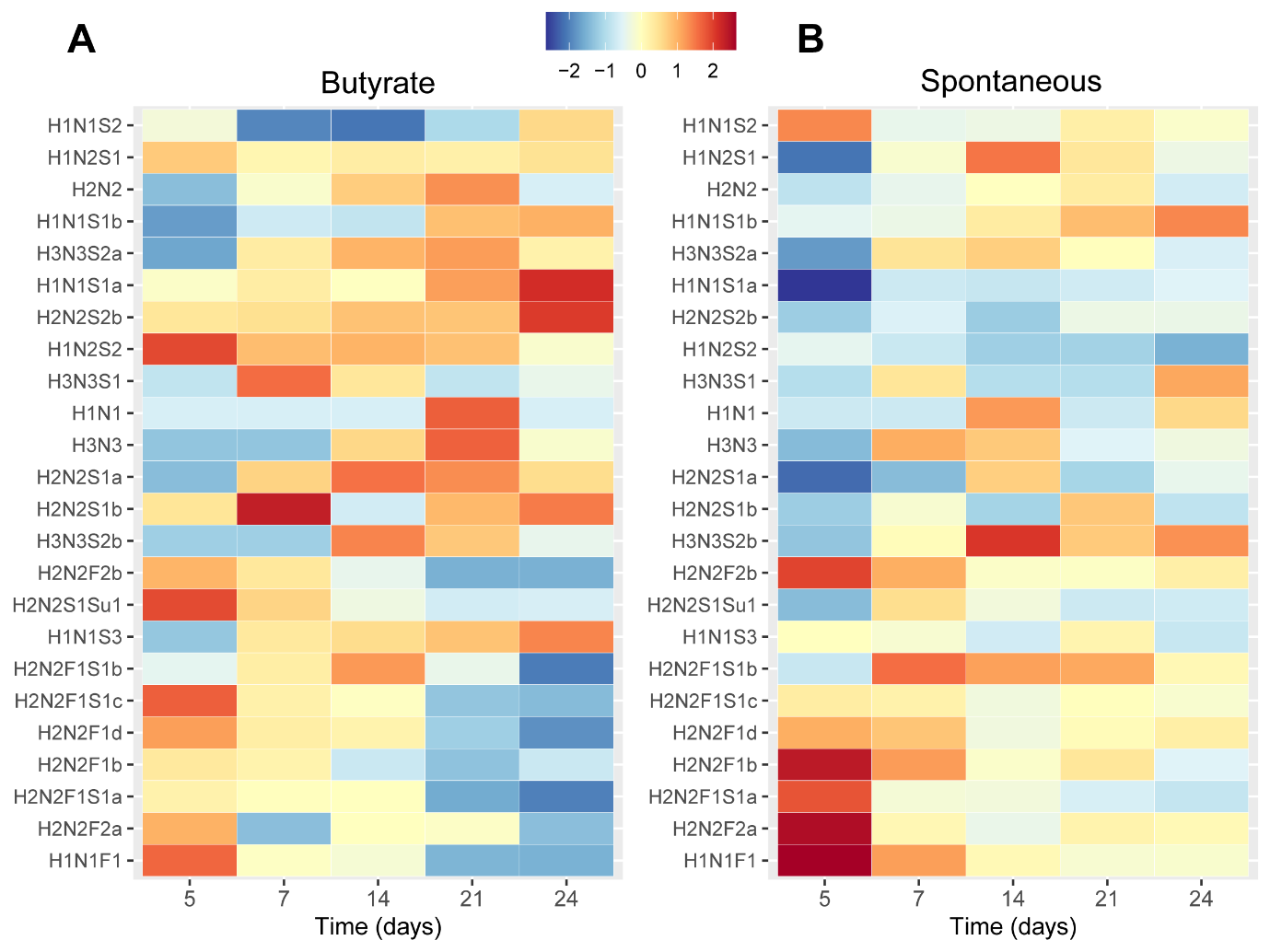

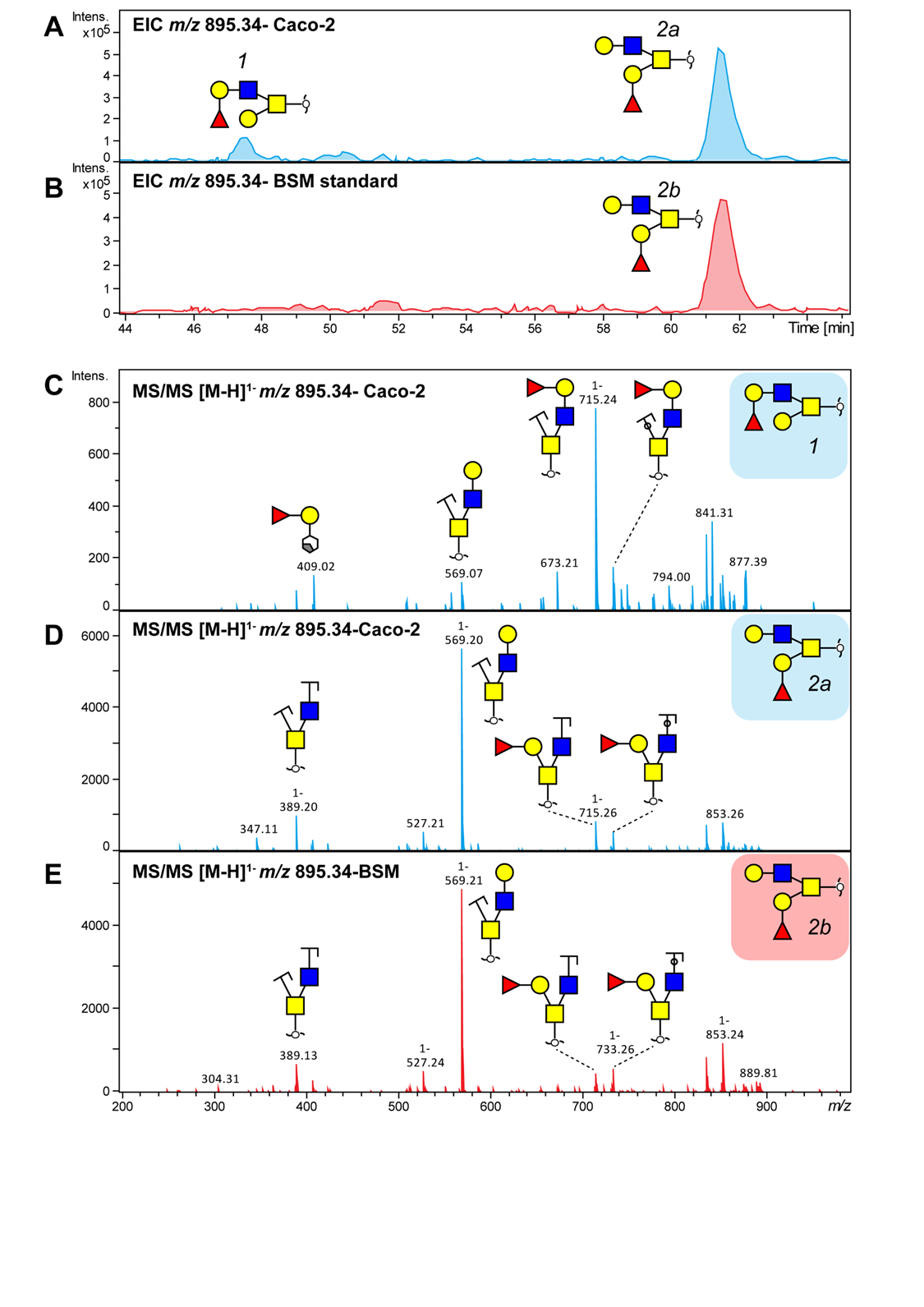


**Supplemental Figure S4. Structural determination of the *O*-glycan isomers with composition H2N2F1. Extracted ion chromatograms (EICs) of *m/z* 895.34 corresponding to the *O*-glycan with composition H2N2F1 in the (a)** CaCo-2 cell line sample and **(b)** bovine submaxillary mucin (BSM) standard. The CaCo-2 cell line sample reveals two isomeric species while the BSM standard contains a single *O*-glycan. The structures of the  isomers eluting at 47.5 and 61.8 min could be assigned based upon the obtained MS/MS spectra. **(c)** The presence of the characteristic cross-ring fragment (*m/z* 409.02) indicates a type 2 blood group H antigen on the 6-arm, which is further supported by the abundant Z ion at *m/z 715.2* for the first isomer in the CaCo-2 cell line. **(d** and **e)**The presence of the Z fragment ions at *m/z* 389.2 and 569.2 indicate occupancy on the 6-arm, while carrying a terminal α1,2-linked fucose to the galactose on 3-arm. This isomer has been identified previously as the major isomer in BSM by NMR [1,2]


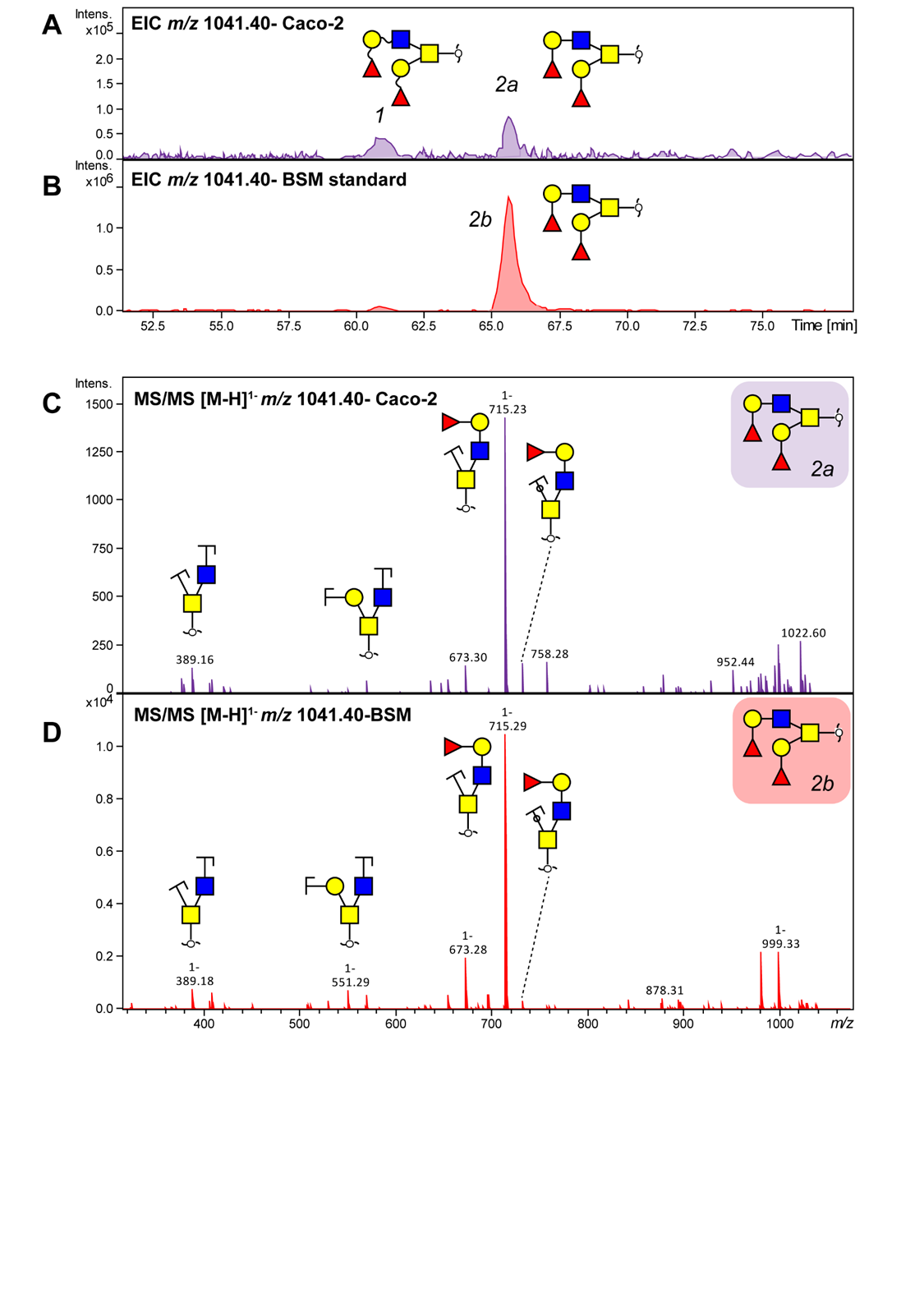


**Supplemental Figure S5. Structural determination of the *O*-glycan isomers with composition H2N2F2.** Extracted ion chromatograms (EICs) of *m/z* 1041.40 corresponding to the *O*-glycan with composition H2N2F2 in the **(a)**  CaCo-2 cell line sample and **(b)** bovine submaxillary mucin (BSM) standard. The Caco-2 cell line sample reveals two isomeric species while the BSM standard contains a single *O*-glycan. The structure of the isomer eluting at 65.5 min could be assigned based upon the obtained MS/MS spectra. **(c** and **d)** The presence of the characteristic Y- and Z-fragment ion pairs at *m/z* 733.3 and 715.3, respectively indicate occupancy on the 6-arm, carrying a terminal α1,2-linked fucose on both arms. This isomer has been identified previously as the major isomer in BSM by NMR [1,2] Due to poor quality of the MS/MS spectra, as well as no corresponding isomer in the BSM standard, the linkages in isomer 1 could not be determined with high confidence.


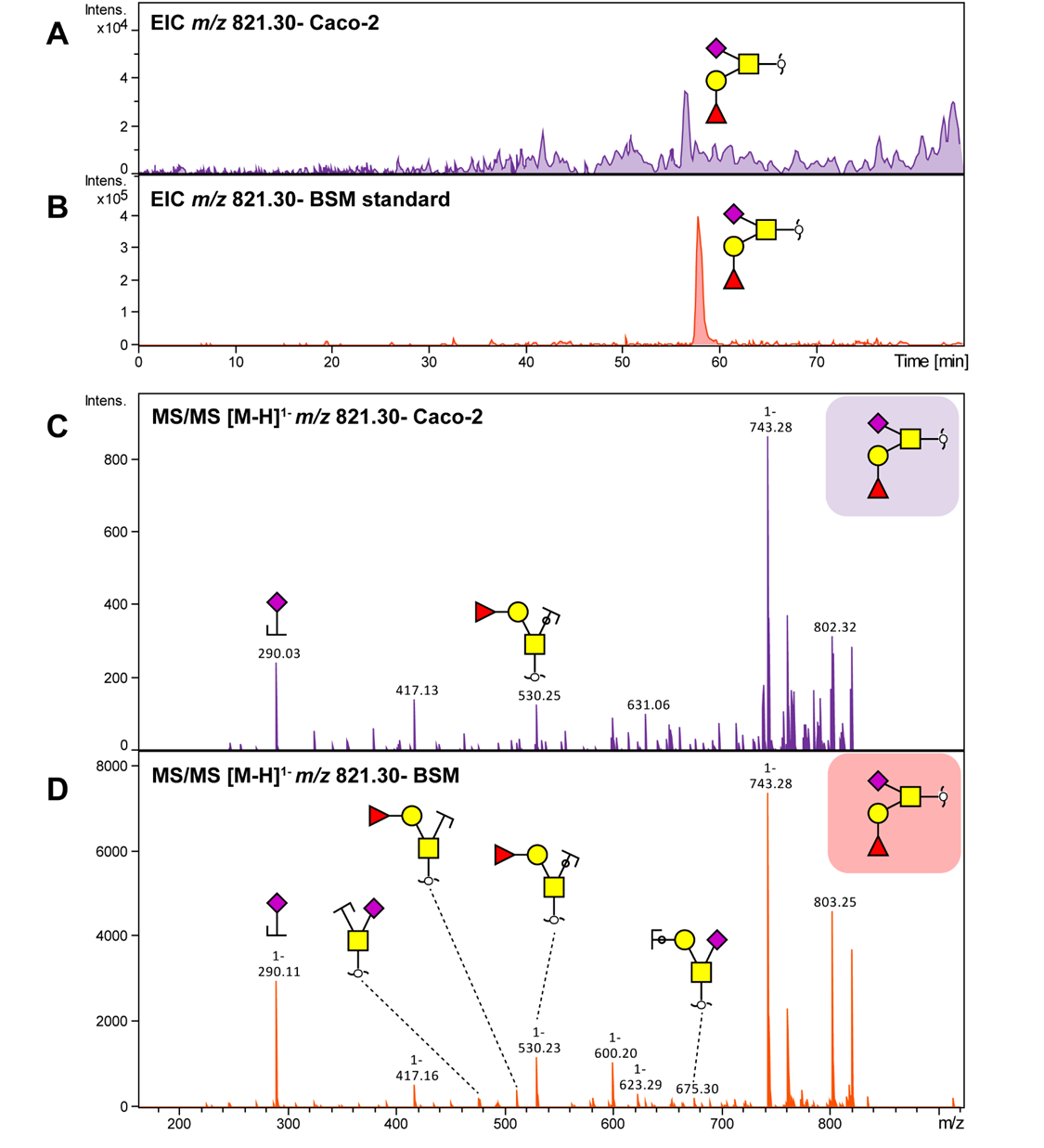


**Supplemental Figure S6. Structural determination of the *O*-glycan with composition H1N1F1S1.** Extracted ion chromatograms (EICs) of *m/z* 821.30 corresponding to the *O*-glycan with composition H1N1F1S1 in the **(a)** CaCo-2 cell line sample and **(b)**bovine submaxillary mucin (BSM) standard. The structure of the  isomer eluting at 59.2 min could be assigned based upon the obtained MS/MS spectra. **(c** and **d)** The presence of the characteristic Y- and Z-fragment ion pairs at *m/z* 512.1 and 530.2, respectively as well as Z-ion at *m/z*495.25 indicate the sialic acid linkage to the innermost *N*-acetylgalactosamine, and a type 3 blood group antigen H on the 3-arm.

*
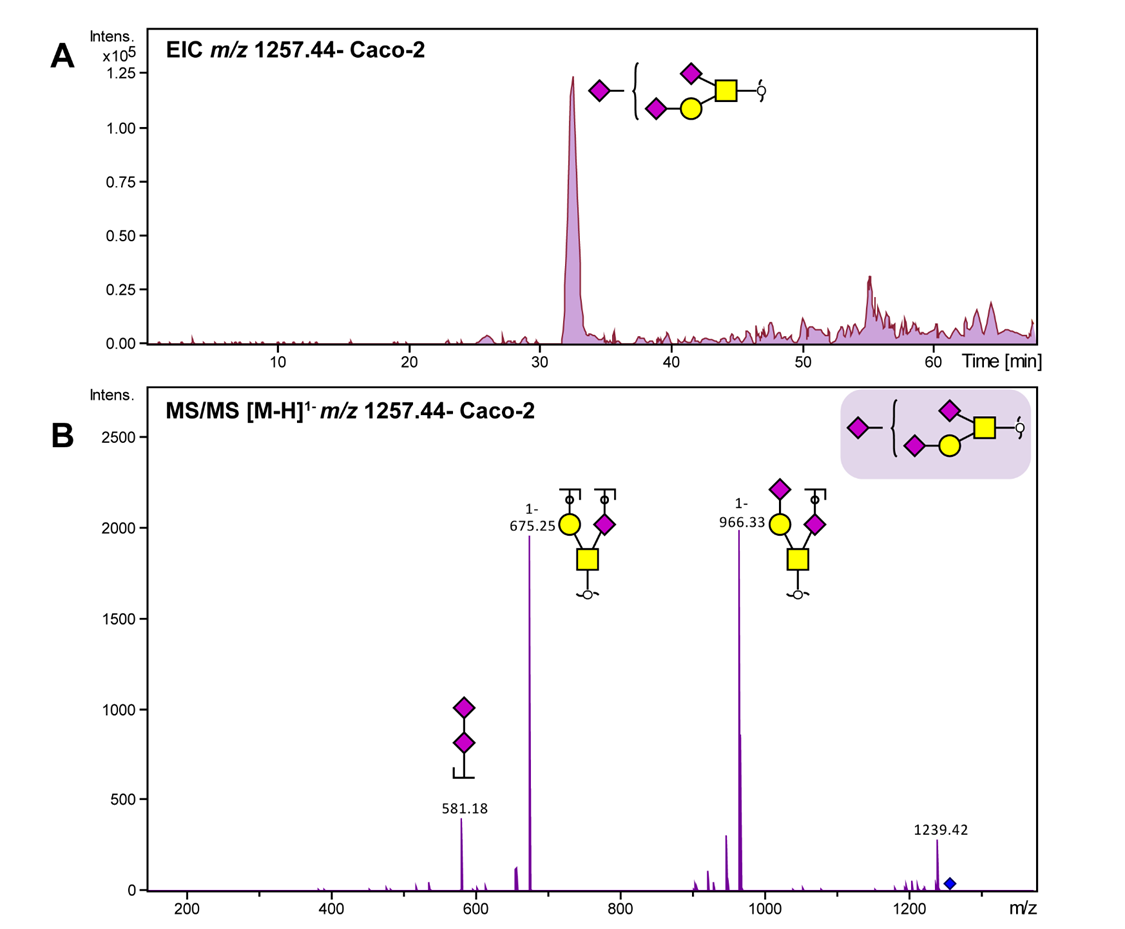
*

**Supplemental Figure S7. Structural determination of the *O*-glycan with composition H1N1S3. (a)**Extracted ion chromatogram (EICs) of*m/z* 1257.44 corresponding to the *O*-glycan with composition H1N1S3 in the CaCo-2 cell line sample. **(b)** The structure eluting at 33.2 min was assigned based upon the obtained MS/MS spectra. The presence of the characteristic B-ion at *m/z* 581.2 indicate the presence of two sialic acids linked together. It could not be determined whether the two sialic acids are present on the 6-arm or the 3-arm of the glycan.

**Supplemental Figure S8. Structural determination of the *O*-glycan carrying the Cad antigen. (a)** Extracted ion chromatogram (EICs) of *m/z* 1169.42 corresponding to the *O*-glycan with composition H1N2S2 in CaCo-2 cell line.**(b)** The structure of the isomer could be assigned based upon the obtained MS/MS spectra. The presence of the characteristic YY-fragment ion *m/z* 675.15 indicate the presence of a core 1 instead of a possible alternative core 3 structure. Additionally, presence of a Z-ion indicating the loss of terminal HexNAc indicates the presence of a Sda antigen. Since the Sda antigen is directly linked to the reducing end GalNAc carrying a sialic acid, it has been assigned as the Cad antigen (GalNAcβ1-4(Neu5Acα2-3)Galβ1-3[Neu5Acα2-6]GalNAc).


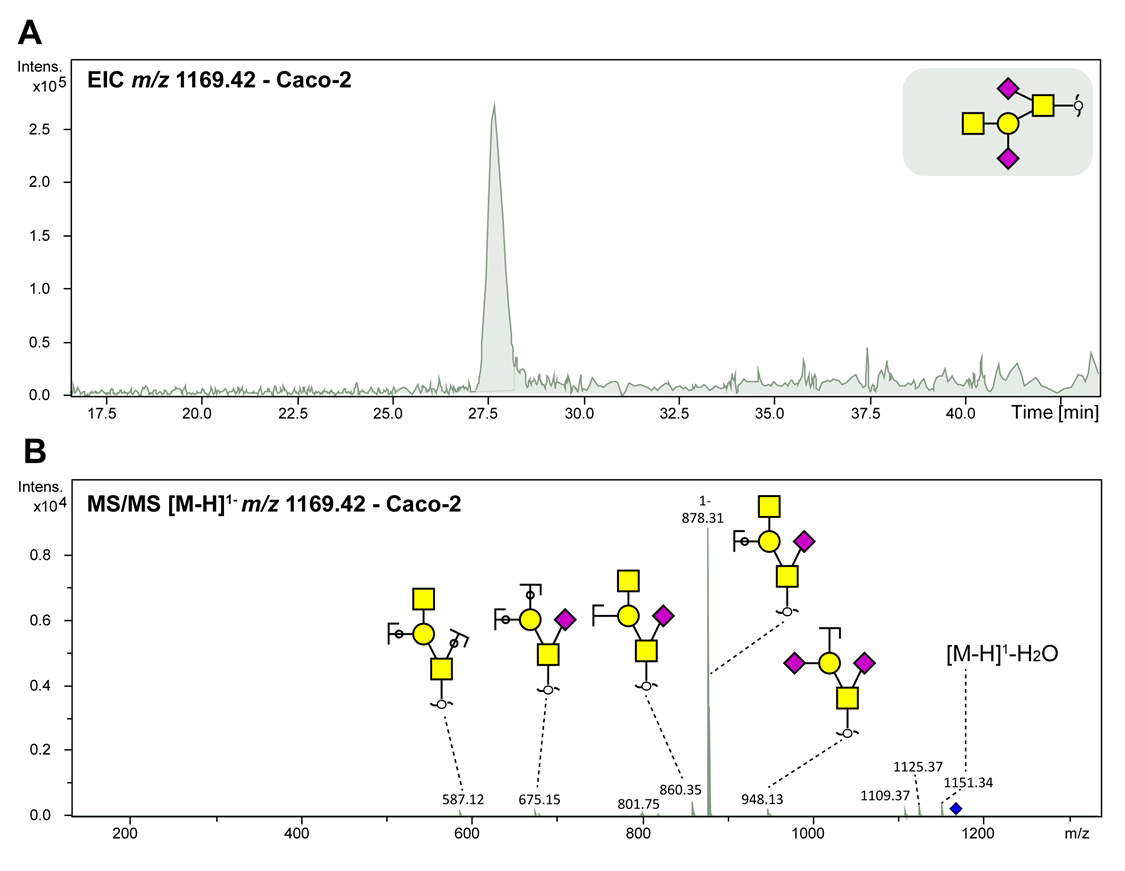


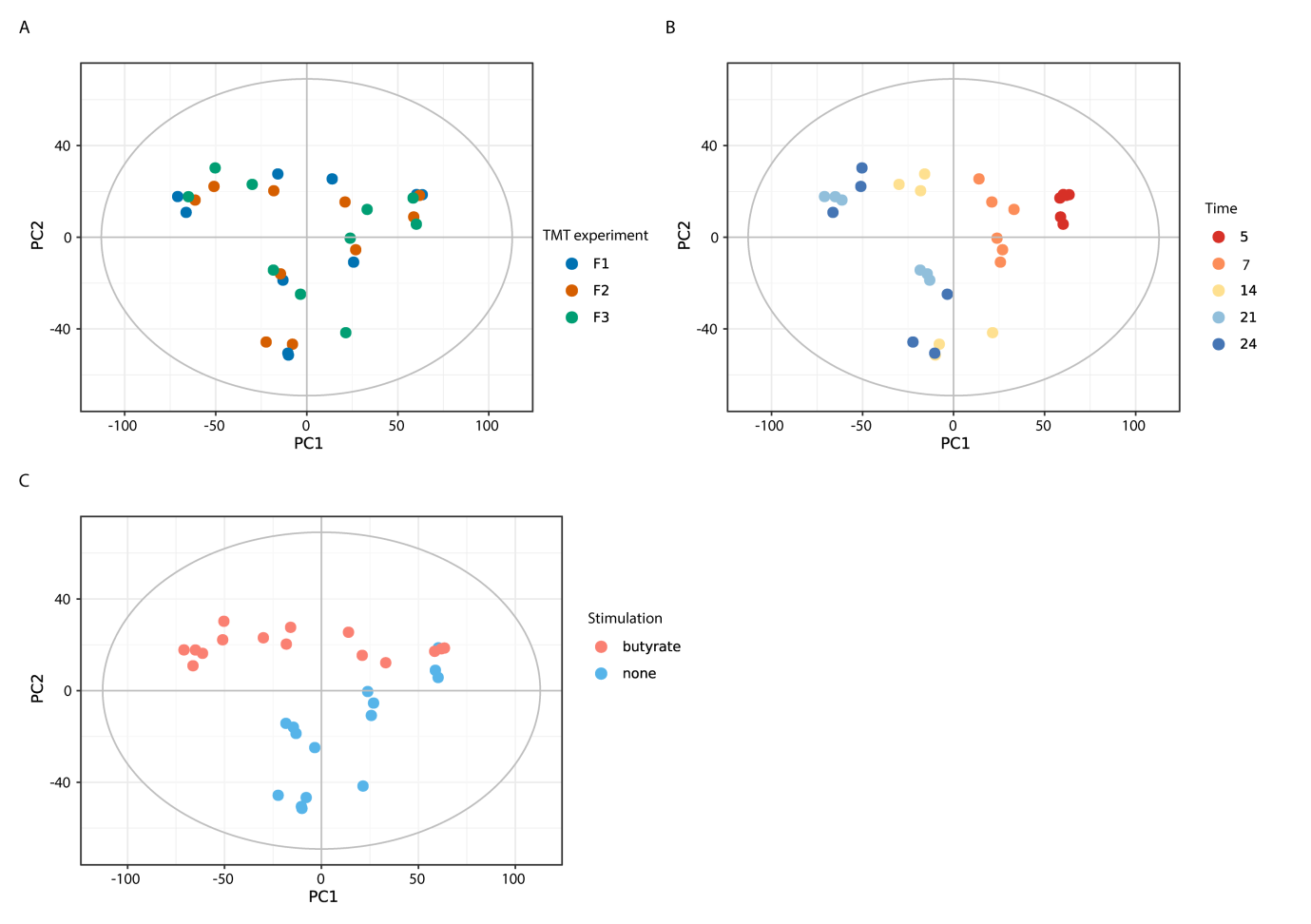


**Supplemental Figure S9. PCA model based on abundances of proteins. (a)** PCA scores plot with three batches (TMT experiment), **(b)** per timepoint in days (5, 7, 14, 21, and 24 day) and **(c)** per condition (butyrate/none). The top two principal components (PC) explain 51.57% of the variation within the data.

**Supplemental Figure S10. Enrichment analysis of protein sets. a)** Protein sets that show a statistically significant change with time (differentiation); **b)** Protein sets that show a statistically significant change with butyrate stimulation. The functional enrichment analysis was performed using g:Profiler (version e106_eg53_p16_65fcd97) with g:SCS multiple testing correction method applying significance threshold of 0.05 [3]

**A**


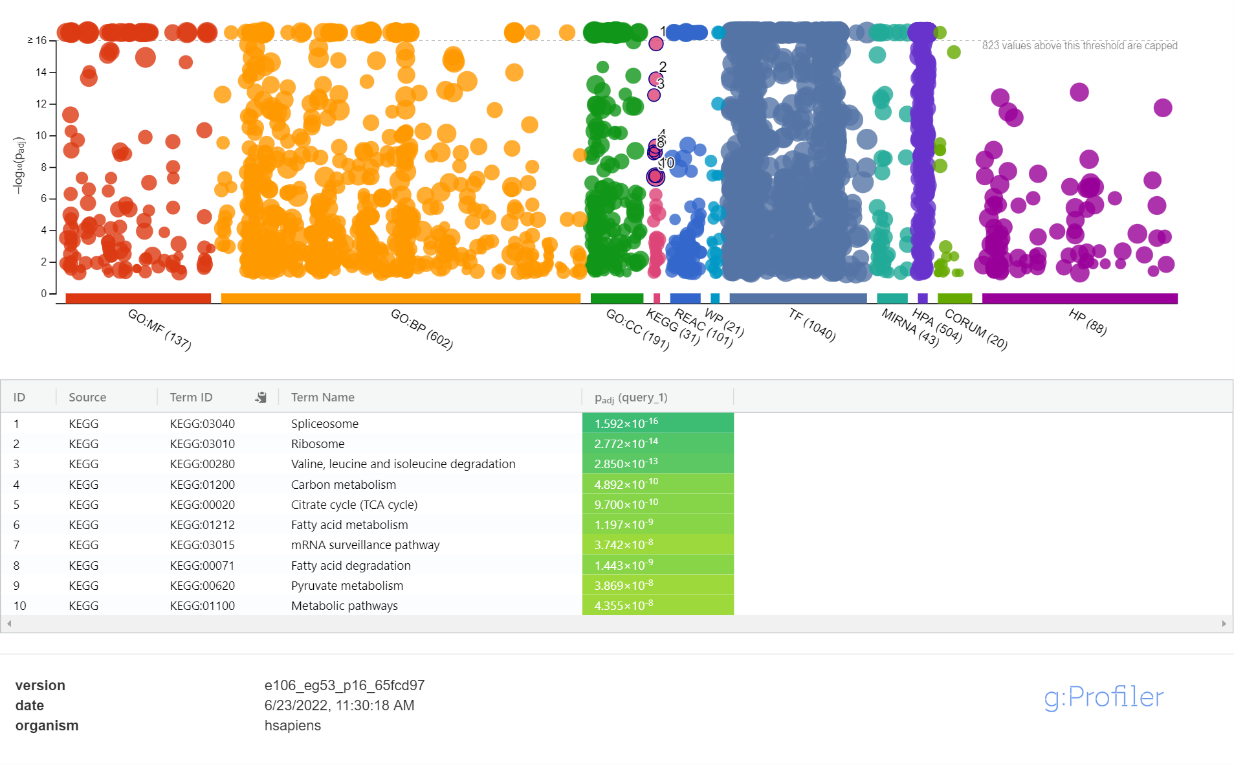


**B**


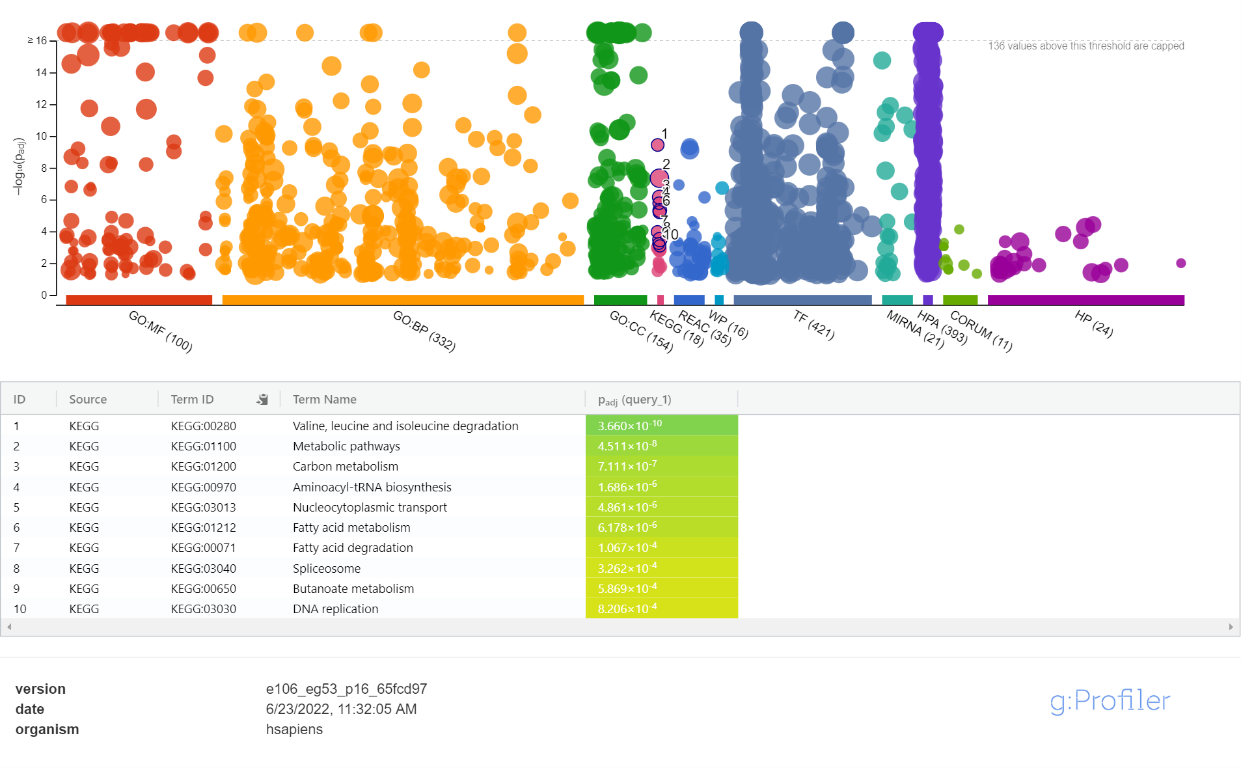


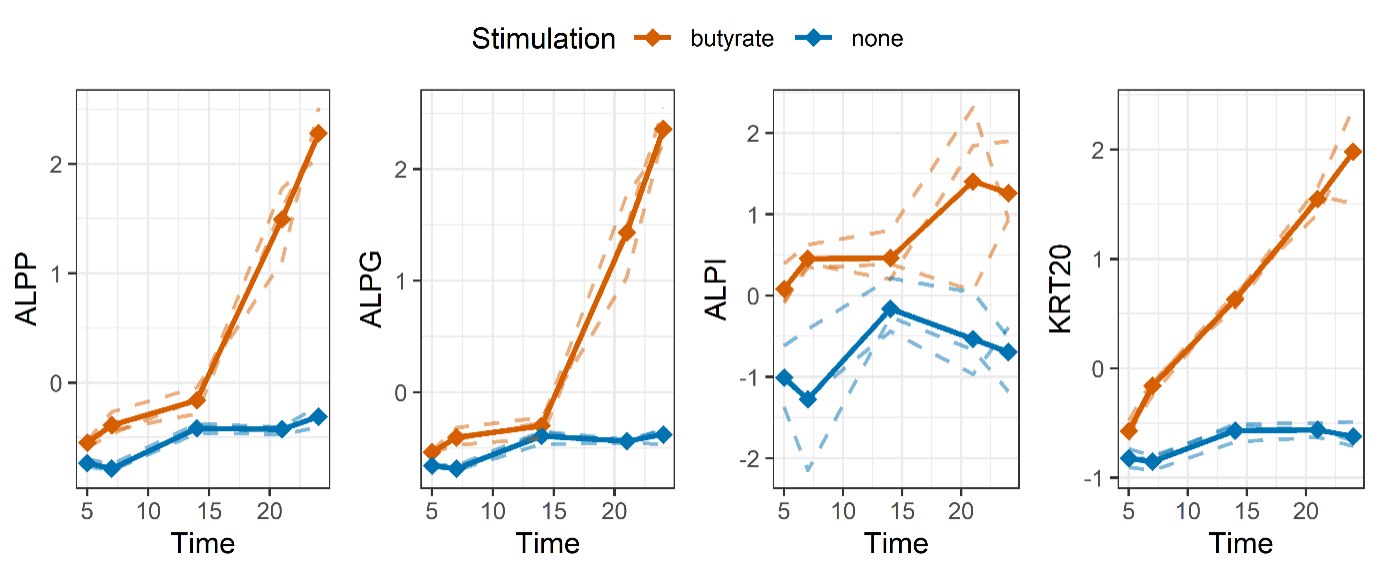


**Supplemental Figure S11. Epithelial cell differentiation markers abundance change with differentiation.** Spaghetti plots of the glycan abundances that show significant difference between timepoints selected from the analysis of variance (ANOVA). The dashed lines represent theZ-scores of the measured values of each biological replicate, whilst the continuous lines represent the Z-scores of the mean values per biological replicate.


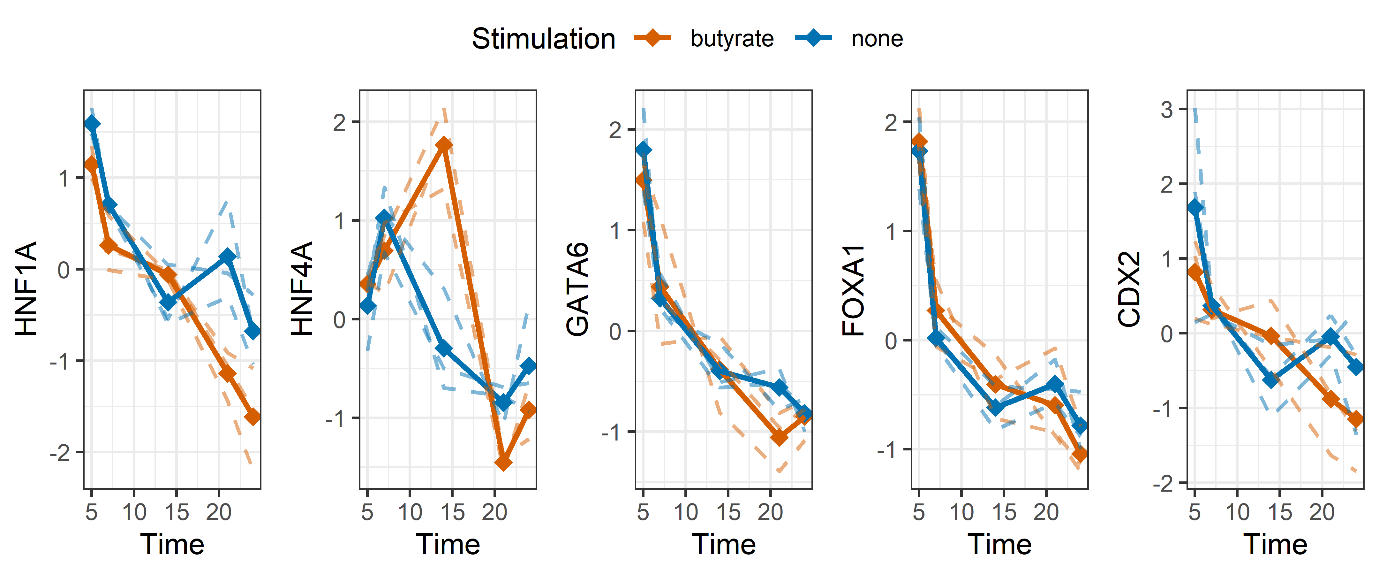


**Supplemental Figure S12. Selected transcription factors abundance change with differentiation.** Spaghetti plots of the glycan abundances that show significant difference between timepoints selected from the analysis of variance (ANOVA). The dashed lines represent the scaled Z-scores of the measured values of each biological replicate, whilst the continuous lines represent the Z-scores of the mean values per biological replicate.


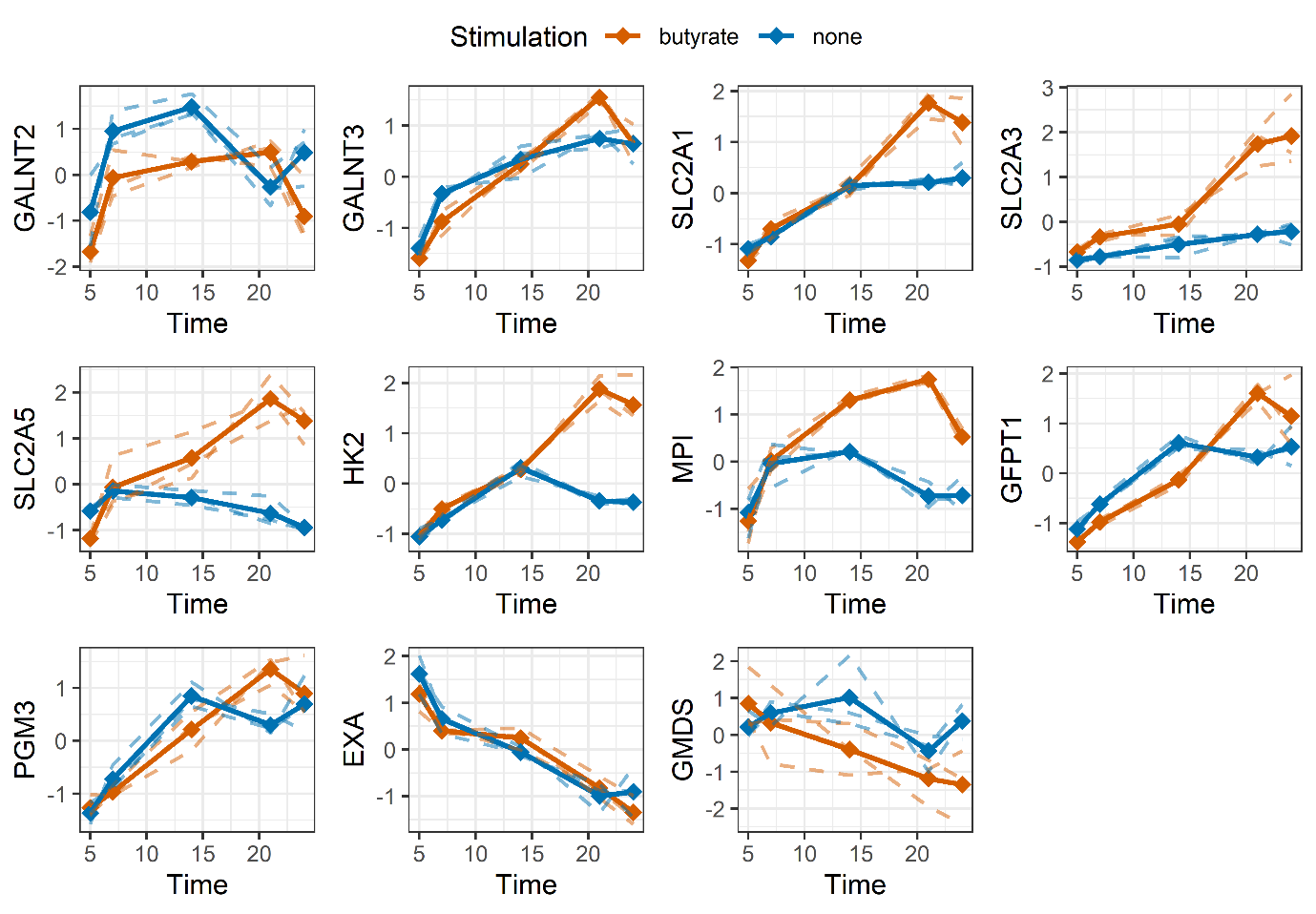


**Supplemental Figure S13. Proteins involved in glycosylation that change with differentiation.** Spaghetti plots of the glycan abundances that show significant difference between timepoints selected from the analysis of variance (ANOVA). The dashed lines represent the scaled Z-scores of the measured values of each biological replicate, whilst the continuous lines represent the Z-scores of the mean values per biological replicate.


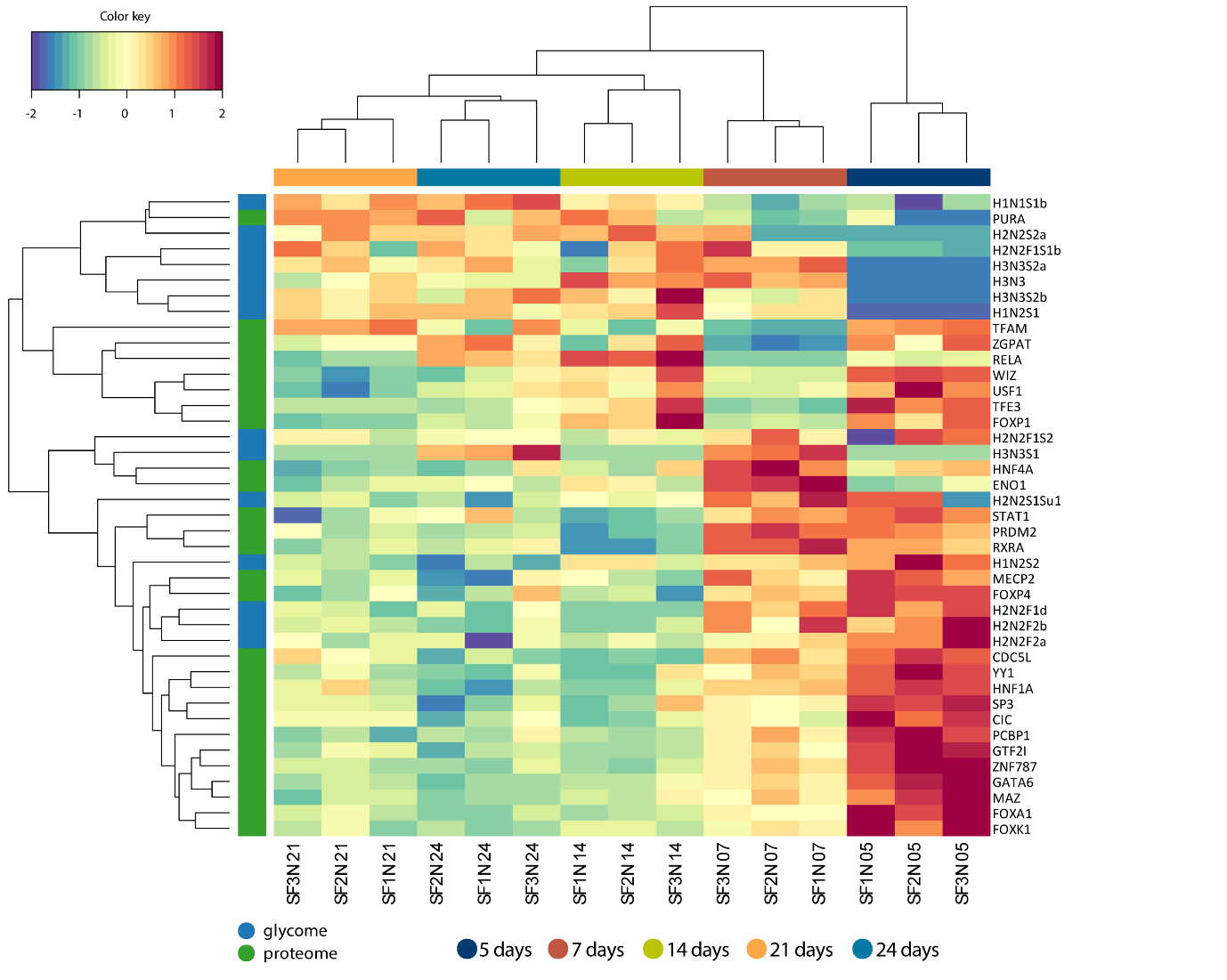


**Supplemental Figure S14. Associations between transcription factors and *O*-glycans for spontaneous differentiation.** Clustered Image Map illustrating changes in both proteome and glycome with differentiation based on a combination of the PLS (Partial Least Square Regression) generalized for the multiple matched datasets and LASSO (least absolute shrinkage and selection operator) based variable selection. The model was tuned for the maximal correlation.


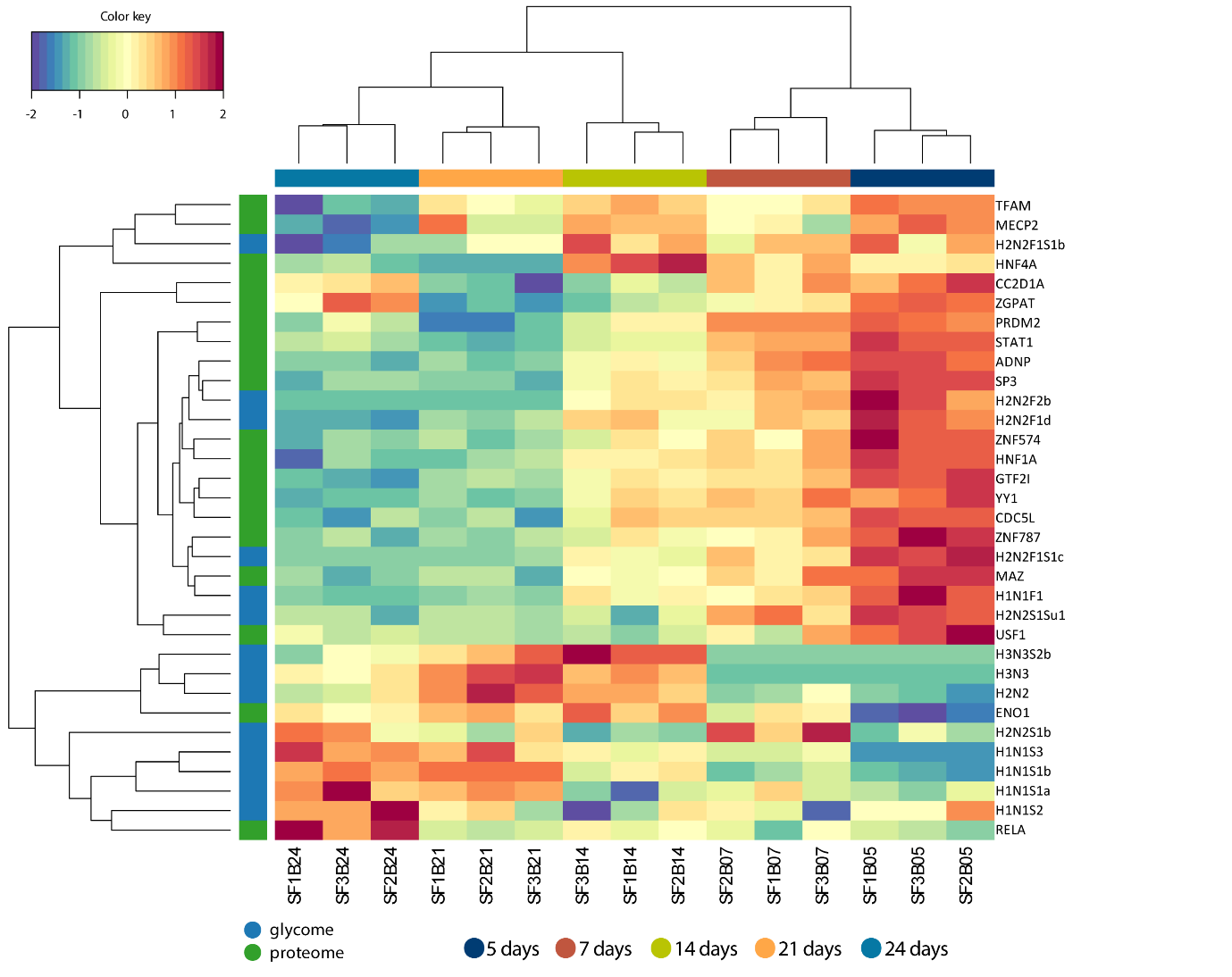


**Supplemental Figure S15. Associations between transcription factors and *O*-glycans for butyrate stimulated differentiation.** Clustered Image Map illustrating changes in both proteome and glycome with differentiation based on a combination of the PLS (Partial Least Square Regression) generalized for the multiple matched datasets and LASSO (least absolute shrinkage and selection operator) based variable selection. The model was tuned for the maximal correlation.

## References

[1] Savage, A. V., S. M. T. D’Arcy, and C. M. Donoghue. 1991. “Structural Characterization of Neutral Oligosaccharides with Blood Group A and H Activity Isolated from Bovine Submaxillary Mucin.” Biochemical Journal 279 (1): 95–103.

[2] Savage, A,V.,Donohue J.J, KoelemanC. A. M., and van den EijndenD. H.. 1990. “Structural Characterization of Sialylated Tetrasaccharides and Pentasaccharides with Blood Group H and Lex Activity Isolated from Bovine Submaxillary Mucin.” European Journal of Biochemistry / FEBS 193 (3): 837–43.

[3] Raudvere, U., Kolberg, L., Kuzmin, I., Arak, T., Adler, P., Peterson, H., &Vilo, J. 2019.“g:Profiler: a web server for functional enrichment analysis and conversions of gene lists (2019 update)”. Nucleic Acids Research, 47(W1), W191–W198.
